# Succession of Particle‐Attached and Free‐Living Microbial Communities in Response to the Degradation of Algal Organic Matter in Lake Taihu, China

**DOI:** 10.1111/1758-2229.70094

**Published:** 2025-04-20

**Authors:** Jing Chen, Yongqiang Zhou, Yunlin Zhang, Quanzhong Guo, Shulan Zhang, Guanghuan Ge, Wenting Jin

**Affiliations:** ^1^ College of Chemistry and Environment Ankang University Ankang Shaanxi China; ^2^ Nanjing Institute of Geography and Limnology, Chinese Academy of Sciences Nanjing China; ^3^ University of Chinese Academy of Sciences Beijing China

**Keywords:** cyanobacterial bloom, free‐living bacteria, high‐throughput sequencing, organic matter, particle‐attached bacteria

## Abstract

Decomposition of Cyanobacterial blooms frequently occurs in Lake Taihu, releasing various fractions of algal organic matter into the water through cell lysis. These fractions influence the production and consumption of dissolved organic matter, nutrient dynamics, and bacterial succession in the lake. However, the interactions between free‐living and particle‐attached bacterial communities with different algal organic matter fractions remain poorly understood. Herein, we investigated the effects of two distinct algal organic matter fractions, obtained through a fractionation procedure simulating cyanobacterial bloom collapse, on freshwater bacterial communities. The degradation of both fractions resulted in stage‐specific changes in the chemical properties of lake water, which were divided into two distinct stages (labeled Stage I and Stage II). *Flavobacteriaceae* was dominant in Stage I, whereas *Methylophilaceae* dominated Stage II. Long‐term ecological observations indicated that particle‐attached bacteria responded more sensitively to different algal organic matter fractions than free‐living bacteria. Compared to the degradation of algal‐derived filtrate, the breakdown of algal residual exudative organic matter led to a more complex free‐living bacterial community network. These findings provide new insights into the capacity of free‐living and particle‐attached bacterial communities to utilize different algal organic matter fractions and highlight their roles in aquatic ecosystems during the post‐bloom stage.

## Graphical abstract

Investigating microbial degradation processes of algal organic matter using fractionation procedure reveals the differential ecological effects of the two distinct algal organic matter fractions from the same lysed cyanobacterial cells on freshwater bacterial communities. The degradation of different algal organic matter fractions instigated a discrepant and graded shift in the succession of the bacterial community, the relationship between dominant bacterial populations and the chemical properties of water, and the co‐occurrence networks of bacterial communities.

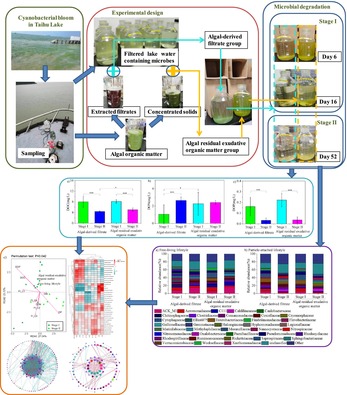

## 1 Introduction

Cyanobacterial blooms result from the massive proliferation and aggregation of cyanobacteria in lakes, reservoirs, and rivers under certain environmental conditions (Morimoto et al. 2020; Wang et al. 2023; Li et al. 2023). Since 1980, cyanobacterial blooms have occurred annually in Lake Taihu, typically from March to November, with the highest cyanobacterial biomass observed between June and September in the northern highly eutrophic part of the lake (Huang et al. 2020; Chen et al. 2022). In recent decades, the extent and coverage of cyanobacterial blooms in Lake Taihu have remained high due to anthropogenic eutrophication and climate change, including slower wind speed and warmer temperatures (Yan et al. 2017; Deng et al. 2018; Gu et al. 2021). As Lake Taihu serves as a drinking water source for medium and large cities in the Yangtze River Delta, dense and toxic cyanobacterial blooms pose significant threats to water quality and supply (Zepernick et al. 2023). The accumulation and deposition of massive bloom biomass in lake sediments following cyanobacterial bloom events significantly burden lake ecosystems (Wu et al. 2019). The collapse and termination of cyanobacterial blooms, accompanied by strong microbial respiration, severely degrade water quality (e.g., elevated nutrient concentrations, reducing pH and dissolved oxygen levels, and releasing dangerous cyanotoxins) and trigger drastic environmental changes, such as the production of foul odor and generation of greenhouse gases (Parveen et al. 2013; Wu et al. 2019; Samudra et al. 2023).

During the post‐bloom stage, cyanobacterial blooms drift across different areas of Lake Taihu and may even form aggregated cyanobacterial biomass in certain bays due to the lake's large surface area. The subsequent decay of these massive blooms in stagnant bays—driven by viral infections and bacterial activity under prolonged sunny conditions—can lead to the rapid release and degradation of algal organic matter, dissolved oxygen depletion, reduced water clarity, and aquatic organism mortality (Shao et al. 2014; Zhou et al. 2015; Yuan et al. 2024). Microbial communities in the lake water and sediment facilitate the decomposition and decolorization of cyanobacterial blooms without human intervention (Han et al. 2022). This suggests that viral infections and bacterial attacks may contribute to the lysis and death of bloom‐forming cyanobacteria. Algal organic matter consists of various organic components, including polysaccharides, proteins, peptides, amino acids, and fatty acids (Her et al. 2004; Han et al. 2016). It is classified into three categories: cellular organic matter (which includes cell debris), extracellular organic matter (rapidly released through algal exudation in a soluble organic form), and intracellular organic matter (internal organic substances released by the rupture of algal membranes) (Du et al. 2022). Among these, intracellular organic matter accounts for 80% of the total algal organic matter during the decomposition of cyanobacterial biomass (Lee et al. 2018). Therefore, it is essential to assess how the compositions of different algal organic matter fractions change after a cyanobacterial bloom to better understand the release of algal organic matter during bloom decline and its effects on microbial proliferation in lake water.

Microbial communities play a crucial role in decomposing, transforming, and consuming algal organic matter, thereby increasing energy fluxes in the biogeochemical cycling of lakes (Bai et al. 2017). In freshwater ecosystems, aquatic microbes are typically classified as either free‐living or particle‐attached microbes (Ren et al. 2023). Previous studies have shown that changes in water and sediment quality parameters induced by bloom decomposition can drive variations in bacterial community composition, leading to bacterial succession (Li et al. 2011; Wu et al. 2019). The relative abundances of *Micrococcineae* and *Legionellales* increased following the addition of *Microcystis* biomass, with *Micrococcineae* becoming predominantly enriched during the decomposition process (Li et al. 2011). Changes in the free‐living and particle‐attached microbial communities were associated with variations in chlorophyll‐*a* (Chl‐*a*) concentration, dissolved organic carbon (DOC), dissolved oxygen, and pH, which were primarily influenced by *Microcystis* breakdown. Furthermore, *Microcystis* decomposition induces phylogenetic clustering and structural instability in sediment microbial communities. Synergistic interactions among saprotrophic bacteria (e.g., *Clostridium*) play a key role in algal organic matter mineralization (Wu et al. 2019). Certain bacterial communities have been identified as active participants, specializing in the decomposition of algal cells through distinct mechanisms that should not be overlooked. However, the dynamic succession of particle‐attached and free‐living microbial communities in response to the degradation of different algal organic matter fractions remains poorly understood.

The objectives of this study were to examine the variation in particle‐attached and free‐living microbial community composition during the degradation of different algal organic matter fractions. This work can help improve our understanding of the differing ecological effects of algal organic matter fractions derived from the same lysed cyanobacterial cells on freshwater bacterial communities. We constructed microcosms containing different algal organic matter fractions, obtained through a fractionation procedure, and incubated lake water containing microorganisms for 61 days under uniform environmental conditions. We hypothesized that the degradation of different algal organic matter fractions would have distinct effects on the dynamic succession of the particle‐attached and free‐living microbial communities. The findings of this study provide a more detailed understanding of the roles of free‐living and particle‐attached bacterial communities in aquatic ecosystems during the post‐bloom stage in Lake Taihu.

## 2 Experimental Procedures

### 2.1 Description of Cyanobacterial Bloom Collapse in Lake Taihu

Lake Taihu, located on the Yangtze Delta Plain, is the third‐largest freshwater lake in China. It supplies water for industrial and agricultural purposes and provides drinking water to approximately 30 million residents (Hu et al. 2017; Yang et al. 2022). The water quality of Lake Taihu has deteriorated due to eutrophication, which has persisted since 2007 as a result of rapid economic development and mismanagement of water resources (Yang et al. 2022). A decline in cyanobacterial blooms was observed at the Lanshanzui, Tuoshan, and Wulihuxin Stations from October 2023 to March 2024. Data on Chl‐*a* concentrations and algal cell densities were obtained from the real‐time data release system for the automatic monitoring of Chinese surface water quality (https://szzdjc.cnemc.cn:8070/GJZ/Business/Publish/Main.html), with detailed variations shown in Figure S1.

### 2.2 Sample Collection and Pre‐Treatment

Lake water and fresh cyanobacterial biomass were collected from the shores of Lake Taihu (trestle area) in June 2021, during the aggregate stage of cyanobacterial blooms. The collected cyanobacterial biomass, considered heterogeneous algal organic matter, was stored in a cool box at 4°C and transported to the laboratory within 12 h of collection. Upon arrival at the laboratory, the biomass was concentrated by passing it through a sterile 20‐μm‐pore nylon net (47 mm in diameter; Merck Millipore, Ireland). Visible particles were removed using sterile tweezers to obtain algal organic matter with approximately 90% moisture content. Meanwhile, lake water samples were filtered through sterile 20‐μm bolting silk, retaining the in situ microorganisms in the filtrate. The freeze–thaw method was then employed to separate the algal organic matter into two fractions: extracted filtrates and concentrated solids (Chen et al. 2022). Both fractions were stored in the dark at 4°C. The lake water samples were subsequently used in algal organic matter fraction degradation experiments.

### 2.3 Experimental Design

The cyanobacterial biomass was pre‐treated with 0.5 L of an algal organic matter –sterile water solution (i.e., 0.03 g mL^−1^ fresh weight). The extracted filtrates and concentrated solids obtained from this solution were then resuspended in sterile water until the final volume reached 0.5 L. The concentration of the extracted filtrates and concentrated solids derived from the algal organic matter was adjusted to approximately 1.5 g L^−1^ (fresh weight) to simulate real accumulation conditions along the lakeshore (Chen et al. 2022).

Subsequently, 0.5 L of the extracted filtrates and concentrated solids were added to a 10.9‐L acid‐cleaned glass container containing 9.5 L of filtered lake water with microbes, forming the algal‐derived filtrate (AF) and algal residual exudative organic matter (AREOM) groups. Specifically, AREOM refers to the metabolites excreted from the concentrated solids into the surrounding water. The covers of all containers were carefully loosened, and after replacing the covers, the containers were manually shaken to ensure proper ventilation. All containers were incubated at room temperature (20°C–25°C) in the dark to prevent disruption of phytoplankton photosynthesis, which would occur under natural light conditions in lake water. Each group consisted of three replicates.

During the 61‐day degradation period of the AF and AREOM groups, water samples were collected on Days 0, 2, 8, 20, 40, 52, and 61 to measure changes in DOC, dissolved organic nitrogen (DON), dissolved organic phosphorus (DOP), dissolved inorganic nitrogen (DIN), and dissolved inorganic phosphorus (DIP) levels. To monitor variations in the main dissolved organic matter (DOM) components during degradation, fluorescent DOM (FDOM) was measured on the same days. Additionally, samples for analysing the free‐living and particle‐attached microbial community structures were also collected on Days 0, 2, 8, 20, 40, 52, and 61.

### 2.4 Interpretation of Bacterial Succession Within Time‐Series Comparisons

Based on the quantity and characteristics of DOM, the degradation of algal organic matter can be divided into three stages: DOM rise, DOM decline, and DOM stabilisation (Chen et al. 2020a, 2022). Additionally, the composition and transformation of DOM may drive the synchronous periodic succession of bacterial communities (Chen et al. 2020a; Xie et al. 2020; Wang et al. 2022). However, in this study, the DOM rise stage was inevitably absent due to the artificial disruption of algal cells using the freeze–thaw method. Therefore, the experimental timeline was divided into two stages—Stage I and Stage II—for the microbial degradation of algal organic matter using a fractionation procedure. Subsamples were collected on Days 0, 2, 8, and 20 to analyse the overall bacterial community during Stage I, while additional subsamples were collected on Days 40, 52, and 61 to analyse the bacterial community during Stage II, following the procedure described by Chen et al. (2022).

### 2.5 Sample Analyses

The daily water samples for subsequent analysis of DOC concentration and FDOM components were refiltered through 0.22‐μm‐pore glass microfiber filters (47‐mm diameter, Shanghai Xingya Purification Material Factory, Shanghai, China) pre‐treated at 450°C for 5 h. The samples were then stored in 100‐mL acid‐cleaned brown glass bottles rinsed with Milli‐Q water. Meanwhile, samples intended for later analysis of DIN, DIP, DON, and DOP concentrations were also refiltered through 0.22‐μm‐porosity filters and stored in 4 × 10‐mL pre‐acid‐cleaned and pre‐alkali‐cleaned test tubes with spiral caps. The DOC concentration was determined using a TOC‐L analyser equipped with an ASI‐L autosampler (Shimadzu, TOC‐L CPH, Japan) (Chen et al. 2022). The primary FDOM components were identified using three‐dimensional fluorescence spectroscopy (Hitachi, F‐7000, Japan) combined with parallel factor analysis (PARAFAC) (Chen et al. 2022). Further details on the DOC and FDOM sample analysis are provided in Supporting Information Text S1. The DIN concentration (DIN=NO_3_^−^‐*N*+ NO_2_^−^‐N+ NH_4_^+^‐N) and DIP concentration (PO_4_^3−^‐P) were measured using a continuous flow analyser (San++, SKALAR, Breda, The Netherlands) (Chen et al. 2022). Total dissolved nitrogen and total dissolved phosphorus concentrations were determined via combined persulfate digestion followed by spectrophotometric measurements (Ebina et al. 1983). The concentrations of dissolved organic nutrients were calculated by subtracting the dissolved inorganic nutrient concentrations from the total dissolved nutrient concentrations.

In general, approximately 1 L of sampled water was pre‐filtered through a sterilised 20‐μm‐pore filter to remove particulate impurities. Particle‐attached bacteria, which are attached to particles ≥ 3 μm in diameter, were retained on sterile filters; whereas free‐living bacteria were captured using 0.2‐μm sterile filters (Ren et al. 2023). Subsamples (~500 mL) were filtered through polycarbonate filters with a 3‐μm pore size (47‐mm diameter, Merck Millipore, Bedford, MA, USA) to collect the particle‐attached bacterial communities. After pre‐filtration through the 3‐μm‐pore filters, the free‐living bacterial communities in approximately 500 mL of water were collected by filtering through polycarbonate membranes with a 0.2‐μm pore size (47‐mm diameter, Merck Millipore, Bedford, MA, USA). DNA extraction was performed using the phenol‐chloroform‐isoamyl alcohol method, and 16S rRNA gene sequencing was conducted to determine the composition of the particle‐attached and free‐living bacterial communities (Chen et al. 2020a). The sequence data were deposited in the National Genomics Data Center database under accession number CRA017057. A more detailed description of the methods used to characterise the particle‐attached and free‐living bacterial communities is provided in Supporting Information Text S2.

### 2.6 Statistical Analyses and Visualisation

All statistical analyses and visualisations were conducted using R software version 4.1.0 and Origin software. Venn diagrams were generated using the ggvenn package (v0.1.9). Two alpha‐diversity indices—Chao1 and Shannon—were calculated using the QIIME workflow. Significant differences in the Chao1 and Shannon indices between the AF and AREOM groups across periods for free‐living and particle‐attached bacterial communities were assessed using analysis of variance (ANOVA), followed by the Kruskal–Wallis test (*p* < 0.05). Linear discriminant analysis effect size (LEfSe) tests were performed to identify discriminative taxa (i.e., biomarkers) enriched during specific degradation periods, with a moderate threshold LDA score of 2.0 (Segata et al. 2011). Beta diversity was measured using Bray–Curtis dissimilarity, calculated based on amplicon sequence variant (ASV) relative abundance among sites, and visualised through principal coordinate analysis (PCoA). Analysis of similarities (ANOSIM) was conducted to assess differences between free‐living and particle‐attached communities at different stages for the AF and AREOM groups, as well as differences between AF and AREOM substrate additions at any stage for free‐living and particle‐attached communities. Principal component analysis (PCA) was performed as an unconstrained redundancy analysis (RDA) using the RDA function in R, based on the relative abundance of bacterial families. In this RDA, vectors representing the direction and magnitude of chemical properties of the water (i.e., DOC, DON, DOP, DIN, DIP, and FDOM composition) were calculated using the envfit function. The vector scores were extracted and scaled using the ordiArroeMul function. An ANOVA‐like permutation test was applied using the anova.cca function on the output from a constrained RDA generated with the rda function, employing 1000 permutations in a single step. In brief, distance‐based RDA was used to determine the relationships between dominant bacterial families and the chemical properties of water during the AF and AREOM processes. Spearman's correlation analysis was conducted to assess these relationships, and a heatmap was generated using the heatmap package in R. Cytoscape was used to perform co‐occurrence network analysis of moderately significant positive and negative correlations (*r* > 0.7 and *r* < −0.7, *p* < 0.05) between bacterial families. The co‐occurrence network was generated using the WGCNA package based on the Spearman correlation matrix. In this network analysis, bacterial families were represented by nodes, while edges in the topological graph indicated correlations between families. Topological network properties were calculated using the igraph package and Gephi (v.0.10) (http://gephi.github.io/) software was used to visualise the co‐occurrence network and perform modular analysis. Putative keystone taxa were identified based on the following thresholds: bacterial families with degrees > 8, closeness centrality > 0.15, and betweenness centrality < 0.025 (Berry and Widder 2014).

## 3 Results

### 3.1 DOM Variability During the Degradation of Different Algal Organic Matter Fractions

**FIGURE 1:**
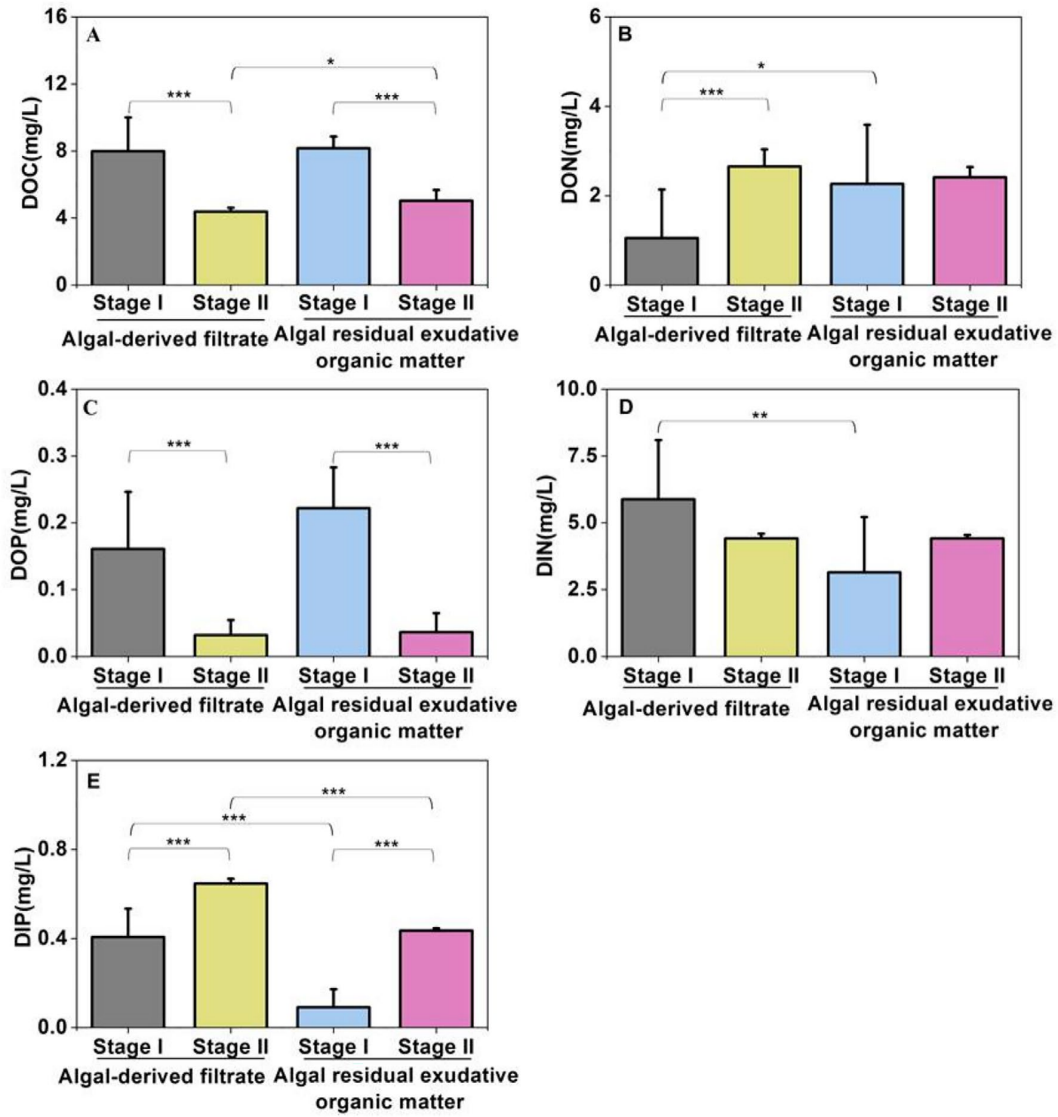
Periodic changes in the dissolved organic carbon (DOC) concentration (A), dissolved organic nitrogen (DON) concentration (B), dissolved organic phosphorus (DOP) concentration (C), dissolved inorganic nitrogen (DIN) concentration (D) and dissolved inorganic phosphorus (DIP) concentration (E) during the 61‐day microbial degradation of algal‐derived filtrate and algal residual exudative organic matter groups. The entire degradation could be divided into two stages: Stage I (0–20 days) and Stage II (40–61 days). Different asterisk letters indicate significant differences between experimental treatments or time stages based on one‐way ANOVA (* indicates *p* < 0.05; ** indicates *p* < 0.01; *** indicates *p* < 0.001).

The DOM concentration and composition in the AF and AREOM groups changed significantly during the 61‐day microbial degradation period (Figure 1; Figure S2). Based on the DOC reduction rate in both groups, the 61‐day degradation period was divided into two stages: Stage I (0–20 days) and Stage II (40–61 days).

The results of a one‐way ANOVA revealed that the DOC concentration in the AF group decreased significantly, from 8.0 ± 2.0 to 4.4 ± 0.2 mg L^−1^ (*p* < 0.001). Similarly, the DOP concentration dropped from 0.2 ± 0.1 mg L^−1^ to below the detection limit (*p* < 0.001) from Stage I to Stage II. In contrast, the mean DON concentration increased significantly from Stage I (1.1 ± 1.1 mg L^−1^) to Stage II (2.7 ± 0.4 mg L^−1^) (*p* < 0.001). Meanwhile, the DIN concentration decreased, while the DIP concentration increased from Stage I to Stage II. In the AREOM group, the mean DOC and DOP concentrations (8.2 ± 0.7 and 0.2 ± 0.1 mg L^−1^, respectively) significantly decreased from Stage I to Stage II (5.0 ± 0.6 mg L^−1^ and nearly zero, respectively) (both *p* < 0.001). However, the mean DON concentration increased only slightly from Stage I (2.3 ± 1.3 mg L^−1^) to Stage II (2.4 ± 0.2 mg L^−1^). The mean DIN and DIP concentrations also increased from Stage I to Stage II.

The DOM fractions exhibited fluorescence properties, reflecting the transformation process of DOM composition during the microbial incubation experiment, referred to as FDOM. The results of PARAFAC identified four independent fluorescence components in the AF and AREOM groups: protein‐like components C1 (λ_Ex/Em_: 225(275–280)/332 nm), C3 (λ_Ex/Em_: 230(275)/320 nm), and C4 (λ_Ex/Em_: 235(305)/348 nm), and a humic‐like component C2 with Ex/Em wavelengths of 265(360)/452 nm. The results of the one‐way ANOVA confirmed that FDOM compositions in the AF group were significantly different between Stages I and II (C1: *p* < 0.001; C2 and C3: *p* < 0.01; C4: *p* < 0.05) (Figure S2). However, in the AREOM group, only the mean fluorescence intensity of the protein‐like C1 component differed significantly between Stages I and II (*p* < 0.001).

### 3.2 Free‐Living and Particle‐Attached Bacterial Community Structures During the Degradation of Different Algal Organic Matter Fractions

After quality trimming, filtering, and the removal of chimeric sequences, a total of 27,033 ASVs were recovered through 16S rRNA amplicon sequencing of the bacterial communities incubated under two different lifestyles during the degradation of various algal organic matter at a 97% similarity threshold.

**FIGURE 2:**
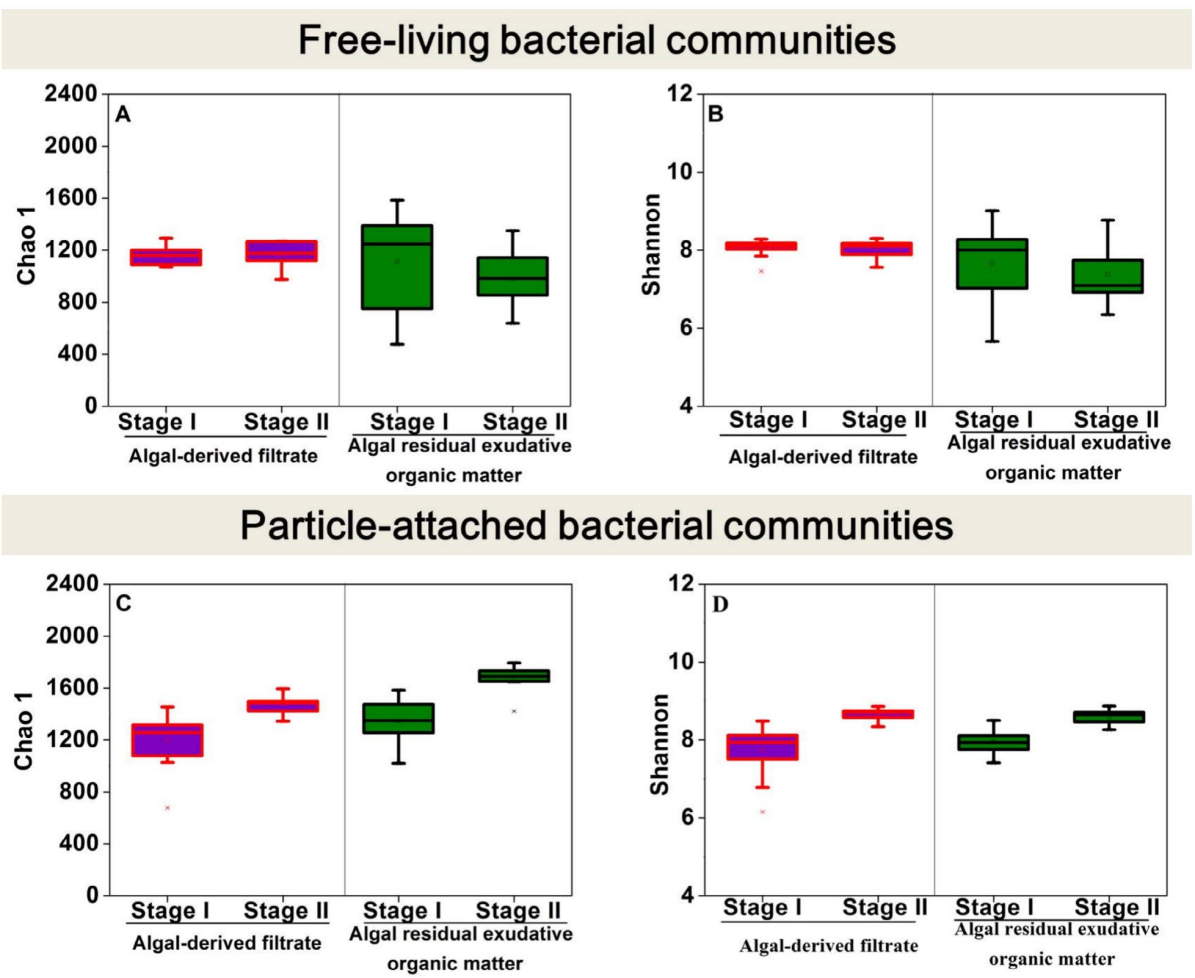
Boxplot of Chao 1 (A, C) and Shannon (B, D) indices in free‐living and particle‐attached bacterial communities at Stage I (0–20 days) and Stage II (40–61 days) for algal‐derived filtrate and algal residual exudative organic matter groups.

The Chao1 index was used to estimate the total number of species in the microbial community at specific time points, while the Shannon index assessed microbial diversity (Yu et al. 2023). In both the AF and AREOM groups, the Chao1 and Shannon indices of the PA microbial community increased significantly from Stage I to Stage II (*p* < 0.05) (Figure 2C; Figure 2D). In contrast, the Chao1 and Shannon indices exhibited only slight variations between stages in either the AF or AREOM group for the free‐living microbial community (Figure 2A; Figure 2B).

**FIGURE 3:**
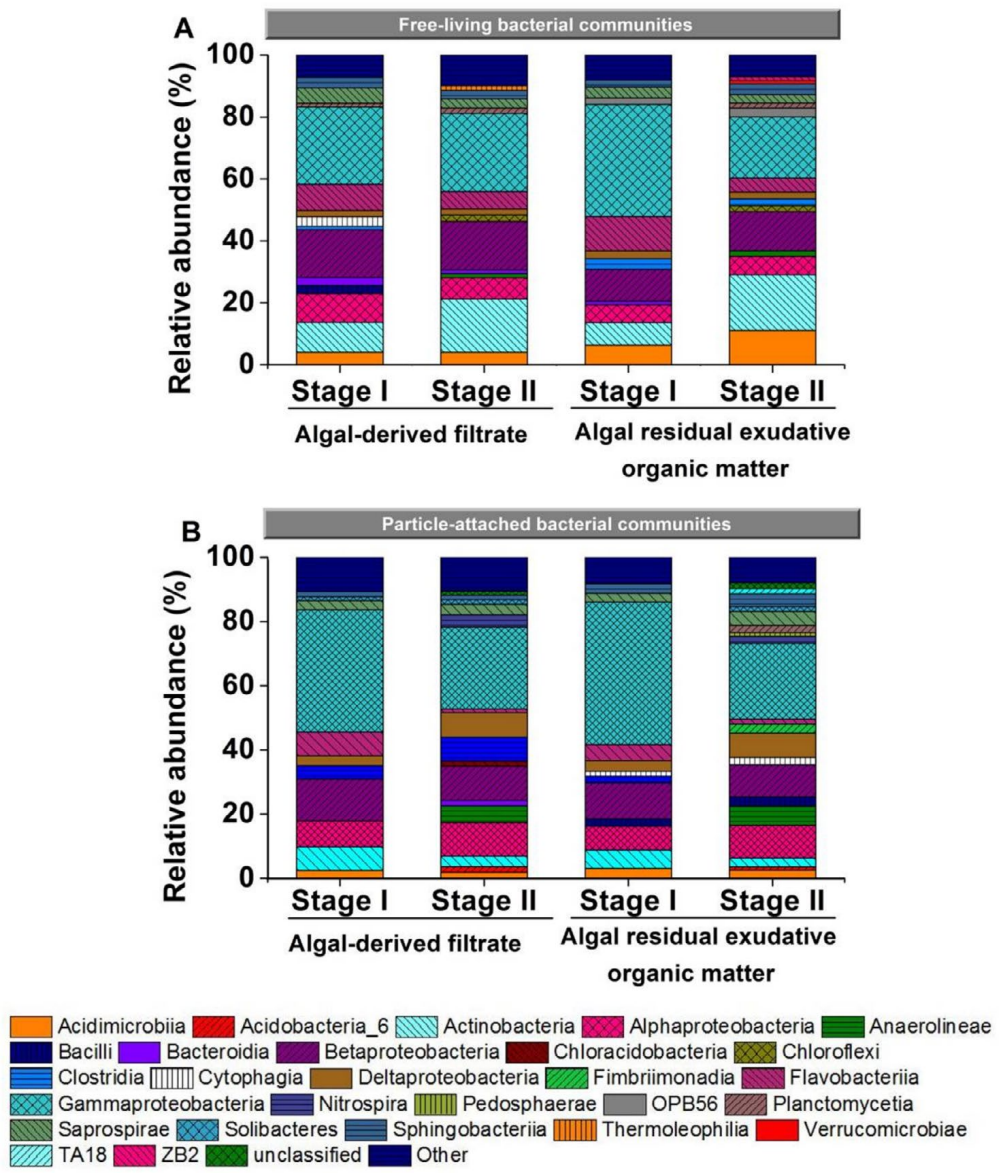
Variations in free‐living (A) and particle‐attached (B) bacterial communities at the class level during the 61‐day microbial degradation of algal‐derived filtrate and algal residual exudative organic matter groups. Others: Sum of taxa with relative abundances < 1%. The entire process can be divided into two Stages: I (0–20 days) and II (40–61 days).

The detected ASVs of the free‐living bacterial communities in both the AF and AREOM groups belonged to 17 main phyla (relative abundance > 1%; Figure 3A). The relative abundance of free‐living *Actinobacteria* significantly decreased from Stage I to Stage II during the microbial degradation of AF and AREOM substrates (both *p* < 0.05), while *Anaerolineae* were detected only at Stage II. In the AF group, the relative abundance of *Alphaproteobacteria*, *Cytophagia*, and *Flavobacteriia* significantly decreased (*p* < 0.05), whereas in the AREOM group, *Gammaproteobacteria* showed a significant decrease from Stage I to Stage II (*p* < 0.05). At the family level, *ACK*_*M1*, *Coxiellaceae*, and *Methylophilaceae* significantly increased (*p* < 0.05), while other free‐living bacteria, such as *Comamonadaceae* and *Flavobacteriaceae*, significantly decreased (*p* < 0.05) in the AF group from Stage I to Stage II (Figure S3A). In the AREOM group, *ACK*_*M1* and *Methylophilaceae* also showed significant increases from Stage I to Stage II (*p* < 0.05).

On the other hand, the detected ASVs of the particle‐attached bacterial communities in the AF and AREOM groups belonged to 16 and 20 dominant phyla, respectively. In both groups, *Gammaproteobacteria* and *Flavobacteriia* decreased significantly from Stage I to Stage II (*p* < 0.05). In contrast, *Deltaproteobacteria* significantly increased from Stage I to Stage II (both *p* < 0.05), while *Anaerolineae* and *Nitrospira* were detected only at Stage II (Figure 3B). In the AREOM group, *Alphaproteobacteria* significantly increased from Stage I to Stage II (*p* < 0.05). Meanwhile, in the AF group, *Comamonadaceae* and *Pseudomonadaceae* decreased significantly (*p* < 0.05) (Figure S3B). In the AREOM group, *Saprospiraceae* significantly decreased, whereas other particle‐attached bacteria, such as *Aeromonadaceae*, *Moraxellaceae*, and *Pseudomonadaceae*, significantly increased from Stage I to Stage II (all *p* < 0.05). The results of LEfSe revealed that particle‐attached *Flavobacteriaceae* and free‐living *Methylophilaceae* were significantly enriched at Stages I and II, respectively, in both groups.

**FIGURE 4:**
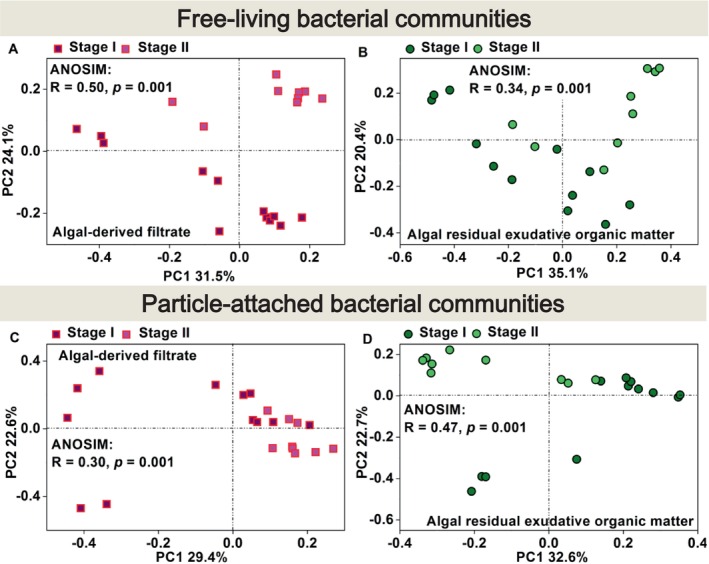
Principal coordinate analysis (PCoA) plot based on the Bray–Curtis distance for free‐living or particle‐attached bacterial communities at stages I and II of the algal‐derived filtrate [free‐living bacterial communities (A); particle‐attached bacterial communities (C)] and algal residual exudative organic matter [free‐living bacterial communities (B); particle‐attached bacterial communities (D)] groups. This shows the classification according to time stage.

To further assess the dynamic variations in the free‐living and particle‐attached bacterial communities during the two stages in the AF and AREOM groups, beta diversity was analysed using PCoA plots based on the ASV level (Figure 4). The free‐living bacterial composition in the AF group varied distinctly between the two stages (ANOSIM: *R* = 0.50, *p* = 0.001). However, the particle‐attached bacterial composition in the AF group showed greater similarity between Stages I and II (ANOSIM: *R* = 0.30, *p* = 0.001). In the AREOM group, the difference between the two stages was greater for the particle‐attached bacteria than for the free‐living bacteria (particle‐attached bacteria ANOSIM: *R* = 0.47, *p* = 0.001; free‐living bacteria ANOSIM: *R* = 0.34, *p* = 0.001).

### 3.3 Relationship Between Bacterial Community and DOM Properties

**FIGURE 5:**
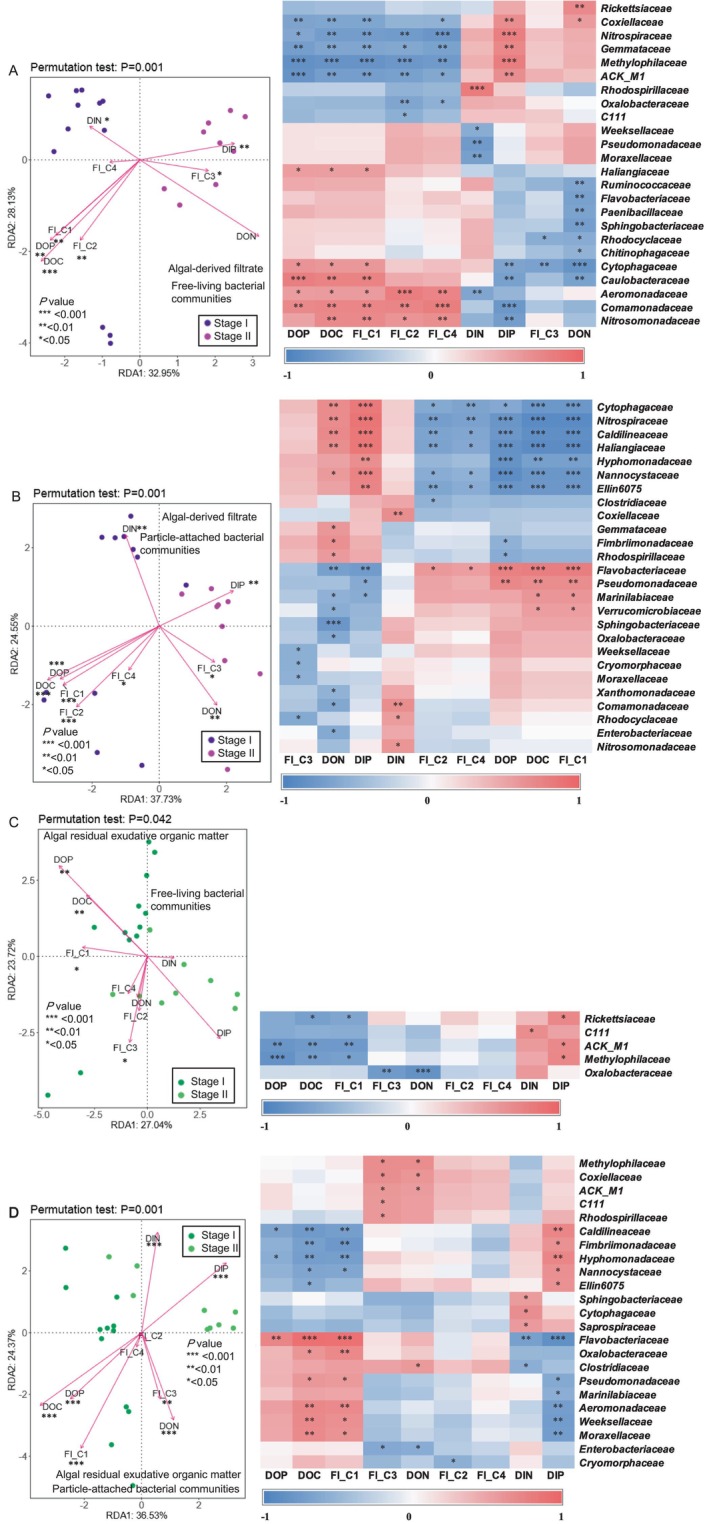
Redundancy analysis and correlation heatmap displaying the relationship between the dominant bacterial families in the algal‐derived filtrate [free‐living bacterial communities (A); particle‐attached bacterial communities (B)] and algal residual exudative organic matter [free‐living bacterial communities (C); particle‐attached bacterial communities (D)] groups and the chemical properties of water during the degradation process.

In terms of lifestyle, the particle‐attached bacteria exhibited a more pronounced response than the free‐living bacteria in the AF group (Figures 5A and 5B). The composition of the free‐living and particle‐attached bacterial communities at Stage I was primarily influenced by DOC, C1 and C2 components, and DOP. In contrast, at Stage II, the primary influences were DON, the C3 component, and DIP. Furthermore, Spearman's correlation analysis revealed that free‐living *Aeromonadaceae* and *Comamonadaceae*, as well as particle‐attached *Flavobacteriaceae*, were significantly positively correlated with DOC, C1, and C2 components, and DOP (all *p* < 0.05). In contrast, free‐living *Methylophilaceae* and particle‐attached *Ellin6075*, *Haliangiaceae*, *Nannocystaceae*, and *Nitrospiraceae* were significantly negatively correlated with DOC, C1, and C2 components, and DOP (all *p* < 0.05). Additionally, free‐living *Cytophagaceae* was significantly negatively correlated with DON, the C3 component, and DIP (all *p* < 0.05). In the AREOM group, DOM properties from Stage I to Stage II explained 50.76% and 60.90% of the variability in the composition of the free‐living and particle‐attached bacterial communities, respectively (Figures 5C and 5D). Specifically, variance in the free‐living and particle‐attached bacterial community compositions was primarily driven by DOC, DOP, and the C1 component at Stage I, while DIP was the main driver at Stage II. Particle‐attached bacteria at Stage II were primarily influenced by DIN. Moreover, free‐living *ACK_M1* and *Methylophilaceae*, along with particle‐attached *Caldilineaceae*, were significantly negatively correlated with DOC, DOP, and the C1 component (all *p* < 0.05). In contrast, particle‐attached *Flavobacteriaceae* showed a significant positive correlation with DOC, DOP, and the C1 component (all *p* < 0.05) and a significant negative correlation with DIP and DIN (both *p* < 0.05). Furthermore, free‐living *ACK_M1* and *Methylophilaceae* were significantly positively correlated with DIP (*p* < 0.05).

### 3.4 Responses of Bacterial Composition to the Degradation of Different Algal Organic Matter Fractions

**FIGURE 6:**
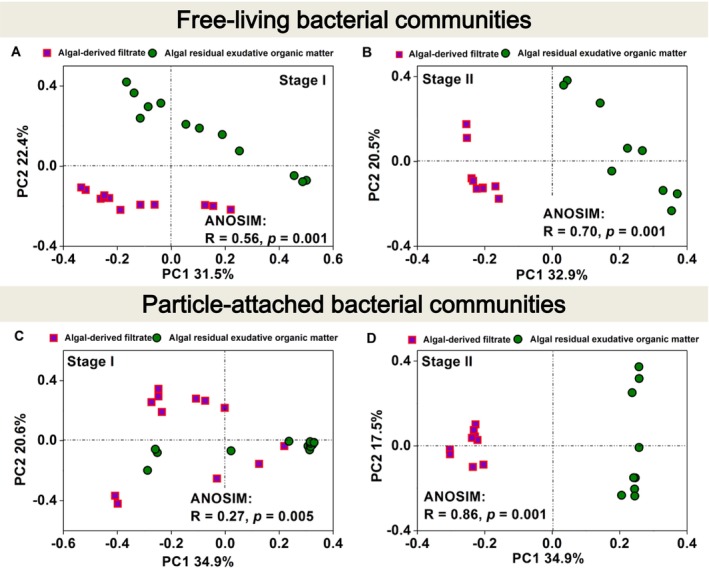
Principal coordinate analysis (PCoA) plot based on the Bray–Curtis distance for free‐living (A, B) and particle‐attached bacterial communities (C, D) at Stages I (0–20 days) and II (40–61 days) of algal‐derived filtrate and algal residual exudative organic matter groups. This shows the substrate classification.

**FIGURE 7:**
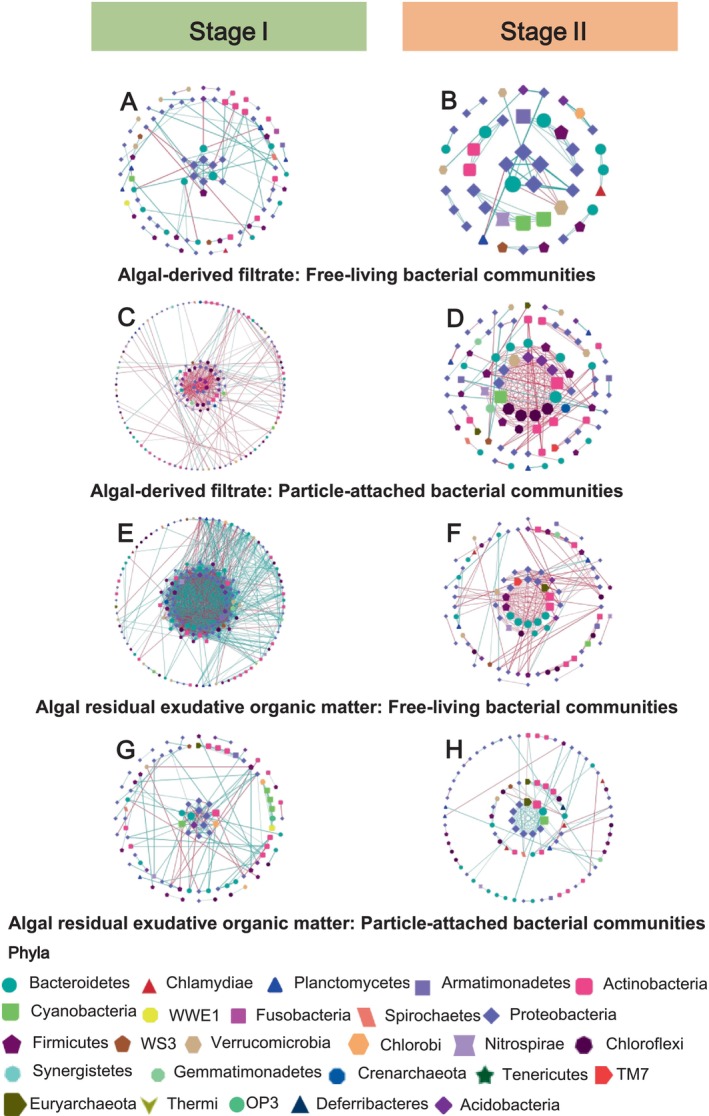
Co‐occurrence networks of the dominant free‐living (FL) or particle‐attached (PA) bacterial families at stage I and stage II of the algal‐derived filtrate [FL‐I (A); FL‐II (B); PA‐I (C); PA‐II (D)] and algal residual exudative organic matter [(FL‐I (E); FL‐II (F); PA‐I (G); PA‐II (H)] groups. Positive and negative correlations are represented by blue and red lines, respectively.

The addition of AF and AREOM had distinct effects on the dynamic succession of bacterial composition (Figure 6). Specifically, free‐living *Comamonadaceae* and *Cytophagaceae*, as well as particle‐attached *Haliangiaceae*, were significantly more abundant in the AF fraction than in the AREOM fraction (*p* < 0.05). The results of PCoA revealed that the addition of the two algal organic matter fractions significantly affected free‐living and particle‐attached bacteria at Stage II but not at Stage I (free‐living bacteria ANOSIM: *R* = 0.56, *p* = 0.001; particle‐attached bacteria ANOSIM: *R* = 0.27, *p* = 0.005) (Figure 6A,C). Moreover, the particle‐attached bacterial community (ANOSIM: *R* = 0.86, *p* = 0.001) exhibited a stronger response to the different algal organic matter fractions than the free‐living bacterial community (ANOSIM: *R* = 0.70, *p* = 0.001) at Stage II (Figure 6B,D). The results of the co‐occurrence network analysis revealed that interactions among free‐living and particle‐attached bacteria in both the AF and AREOM groups were predominantly positive at both stages (Figure 7). The free‐living bacterial networks in the AREOM group were more complex, containing more nodes and edges than those in the AF group at both Stage I and Stage II. Additionally, the particle‐attached bacterial networks in the AREOM group (95 nodes, 163 edges) were smaller than those in the AF group (141 nodes, 337 edges) at Stage I. The sizes of both free‐living and particle‐attached bacterial networks in the AF and AREOM groups decreased from Stage I to Stage II. Weighted average network degree centralities, closeness centralities, and interactive betweenness centralities of free‐living and particle‐attached bacteria were measured in both groups (Table 1). Network analysis identified four main putative keystone particle‐attached families—*Solirubrobacteraceae*, *Acidobacteriaceae*, *Solibacteraceae*, and *FFCH7168*—at Stages I and II in the AF group. In the AREOM group, *Barnesiellaceae* emerged as a putative keystone free‐living and particle‐attached bacterial family at Stage II.

**TABLE 1:** Topological properties of the empirical networks of the free‐living and particle‐attached bacterial communities for algal‐derived filtrate and algal residual exudative organic matter groups.

Group name	Bacterial lifestyle	Stage	Weighted average network degree centrality	Weighted average closeness centrality	Weighted average interactive between centralities
AF	Free‐living bacteria	I	2.52	6.13	29.31
		II	0.98	1.40	2.76
	Particle‐attached bacteria	I	3.07	11.34	98.11
		II	3.53	11.59	98.09
AREOM	Free‐living bacteria	I	3.02	11.49	108.6
		II	1.13	2.75	8.02
	Particle‐attached bacteria	I	2.71	8.82	28.64
		II	0.61	1.46	15.48

## 4 Discussion

Although the microbiological drivers of cyanobacterial bloom decline have been frequently investigated (Wang and Chen 2008; Dai et al. 2018; Chen et al. 2020b), few studies have focused on the microbial degradation of algal organic matter fractions. During cyanobacterial bloom degradation, the large‐molecule outer layer of cyanobacteria deteriorates, the cyanobacterial cell population collapses, and small‐molecule intracellular substances break down (Zhang et al. 2020). Therefore, obtaining molecular weight–fractionated algal organic matter is essential for a better understanding of the mechanisms underlying microbial degradation of cyanobacterial blooms following cell lysis (Zhang et al. 2020; Zorz et al. 2023). We found that stage‐specific changes in water chemistry during algal organic matter degradation drive bacterial community succession, with different fractions of algal organic matter selectively promoting distinct bacterial communities during the post‐bloom phase. Statistical analyses (i.e., PCoA and RDA) and network analysis provided complementary insights into the periodic and substrate‐driven bacterial associations.

### 4.1 Periodic Dynamics of Bacteria Communities

Previous studies have reported periodicity in bacterial community structure during microbially driven algal organic matter degradation (Wang et al. 2022; Chen et al. 2025). In this study, this periodicity was further supported by the microbial degradation of different algal organic matter fractions. The addition of AF led to an increase in the humic‐like component and DOC concentration at the beginning of degradation. Some free‐living bacteria (i.e., *Cytophagia*, *Deltaproteobacteria*, and *Flavobacteriia*) and particle‐attached bacteria (i.e., *Betaproteobacteria*, *Flavobacteriia*, and *Gammaproteobacteria*) in the AF group thrived during Stage I (Figure 3). *Flavobacteriia* has been reported as a key alga‐associated bacterium with a high capacity for algal polysaccharide utilisation (Martin et al. 2016; Kappelmann et al. 2019). In the AREOM group, the release of AREOM inevitably increased the DON concentration and protein‐like components in the water. The addition of algal residues likely introduced alga‐associated bacteria (*Flavobacteriia*) at the beginning of degradation. From Stage I to Stage II, bacterial consumption led to decreases in DOC and DOP concentrations and the accumulation of inorganic nutrients, which promoted an increase in the abundance of free‐living and particle‐attached *Anaerolineae* in the AREOM group. The *Anaerolineae* lineage of *Chloroflex*i has been reported as a core population in anaerobic digesters and is potentially involved in cellular adhesion and cellulose degradation (Xia et al. 2016).

Periodicity was also evident in bacterial alpha and beta diversities (Figures 2 and 4). However, compared to particle‐attached bacteria, free‐living bacteria exhibited less pronounced periodicity in alpha diversity indices (i.e., Chao1 and Shannon indices) in both the AF and AREOM groups. This could be attributed to the consistent availability of substrates provided to free‐living communities across stages (Skouroliakou et al. 2024). PCoA results showed that the difference between the two stages was greater in the AF group than in the AREOM group. This difference has been associated with the addition of AF, which induced more significant variations in DOM concentration and composition than the addition of AREOM (Figures 1 and S2).

### 4.2 Exploring the Relationships Between the Bacterial Community and Chemical Properties of Water

In the AF group, lake water properties—including DOC, DON, DOP, DIP, and C1 and C2 components—were the primary drivers of free‐living and particle‐attached bacterial communities. The lake water properties in the AF group were characterised by high DOC and humic‐like component concentrations, which were up to 0.6 and 6.3 times higher than the values typically observed after the addition of an equivalent algal organic matter (Chen et al. 2022). The higher fluorescence intensity of the humic‐like component in the AF group compared to the AREOM group at Stage I indicates that humic‐like substances are inherent intracellular organic matter released by the rupture of algal cell membranes (Villacorte et al. 2015; Hua et al. 2019; Zang et al. 2020). The family *Comamonadaceae* has been reported as a key bacterial group involved in the biodegradation of recalcitrant polymers in humic lakes and plays a crucial role in nitrogen, phosphorus, and sulphur metabolism (Deja‐Sikora et al. 2019; Taipale et al. 2023; Liu et al. 2024). This is evidenced by the significant positive correlation between the humic‐like C2 component and the abundance of *Comamonadaceae* at Stage I (Figure 5). A high concentration of humic‐like components promotes the growth of free‐living *Comamonadaceae* and potentially contributes to nutrient recycling. Moreover, the accumulation of humic‐like components also supported the survival of free‐living *Comamonadaceae* during the 61‐day equivalent algal organic matter degradation period (Figure S4). Free‐living *Aeromonadaceae* was also prominent at Stage I (Figure S3), and its capability for denitrification—such as the reduction of nitrate to nitrite—has been demonstrated (Uchida et al. 2018), suggesting that *Aeromonadaceae* drives nitrogen metabolism depending on intracellular cyanobacterial organic carbon.

In the AREOM group, lake water properties—including DOC, DOP, DIP, and the protein‐like C1 component—were the primary factors driving the succession of free‐living and particle‐attached bacterial communities. Compared to the free‐living bacterial community, the particle‐attached bacterial community exhibited a stronger correlation with lake water properties during AREOM degradation (Figure 5C,D). This result highlights the dependence of PA bacteria on organic substrates in lake water. For example, particle‐attached *Caldilineaceae*, which thrives during Stage II, showed significant negative correlations with DOC, DOP, and the protein‐like C1 component, suggesting that these bacteria specialise in consuming labile DOP. Most members of *Caldilineaceae* are well‐adapted to anaerobic hydrolysis and phosphorus removal in wastewater treatment systems (Liu et al. 2020; Carles et al. 2022; Zhang et al. 2024a). Kindaichi et al. (2013) suggested that *Caldilineaceae* may contribute to changes in the respiration profiles of other PA bacterial families, thereby enhancing biological phosphorus removal in wastewater treatment plants.

Particle‐attached *Flavobacteriaceae* and free‐living *Methylophilaceae* thrived during Stages I and II, respectively, throughout both AF and AREOM degradation. The dynamics of these two bacterial families were similar to those observed during bulk algal organic matter degradation (Figure S4). *Flavobacteriaceae* are commonly associated with phytoplankton blooms in various aquatic environments and utilise diverse polymer‐degrading enzymes to break down high‐molecular‐weight polysaccharides and proteins found in algal organic exudates and lysates (Zhu et al. 2023; Juste‐Poinapen et al. 2024; Zhang et al. 2024b). A previous study showed that dominant algal organic matter—comprising both extracellular and cellular organic material—primarily consists of proteins and polysaccharides (Pivokonsky et al. 2014). During algal polymer degradation, *Flavobacteriaceae* convert high‐molecular‐weight compounds into low‐molecular‐weight compounds (Zhu et al. 2023). Moreover, homologues of gliding proteins enriched in *Flavobacteriaceae* play a pivotal role in microbial attachment during algal polymer degradation (Gavriilidou et al. 2020). *Methylophilaceae* are ubiquitous in lakes, with several members of this family potentially responsible for cycling one‐carbon compounds (i.e., formaldehyde, methanol, and methylated sulphur compounds) (Lapidus et al. 2011; Eyice et al. 2015; Yu et al. 2017). Some *Methylophilaceae* have been shown to encode methylotrophic functions, enabling the degradation of dimethyl sulphide in Lake Tocil sediment (Eyice et al. 2015).

### 4.3 Different Algal Organic Matter Fractions Promoted Distinct Bacterial Communities

In this study, AF or AREOM was added as a nutrient substrate, providing a medium for the growth of similar free‐living and particle‐attached microbes at the same stage (Figure S5), indicating that some components of AF were similar to those of AREOM. For example, both AF and AREOM were assumed to contain the C1 component from Stages I to II (Figure S2). Previous studies suggest that the similarity of DOM compositions in soil extract and original coastal water promotes comparable major changes in bacterial community composition and function over a short‐term incubation period (Zhao et al. 2023).

However, it is important to note that the addition of different algal organic matter fractions caused variations in the composition of free‐living and particle‐attached bacterial communities. In particular, free‐living *Comamonadaceae*, *Cytophagaceae*, and particle‐attached *Haliangiaceae* were distinct in the AF fraction compared to the AREOM fraction. Most members of *Comamonadaceae* rely on sulphur‐oxidising metabolism (Deja‐Sikora et al. 2019), which may explain the predicted higher dissolved sulfidic content in the AF fraction. *Cytophagaceae* are identified as heterotrophic bacteria capable of digesting polysaccharides or proteins secreted by cyanobacterial cells (Zhu et al. 2021). *Haliangiaceae* are specialised decomposers of macromolecules such as starch, proteins, and casein (Ivanova et al. 2010). These differences are likely due to the higher dissolution of polysaccharides, proteins, starch, and casein in the AF fraction compared to the AREOM fraction. Particle‐attached bacteria with high extracellular enzyme activity generally exhibited a greater advantage than free‐living bacteria under conditions of higher organic carbon availability (Cao et al. 2016, 2020). The co‐occurrence networks of free‐living bacterial communities in the AREOM group were more complex than those in the AF group, with the key bacterial families in the networks of the two groups being completely different (Figure 7; Figure S6). In particular, at Stage I, compared to AF, the co‐occurrence network for AREOM exhibited a larger size (total nodes), greater connectivity (total links), and higher average connectivity (weighted average network degree centralities), along with a greater number of ecological niches (modules, i.e., major ecological clusters) for free‐living bacterial communities. This was likely due to the improved resource availability of AREOM, which fostered stronger free‐living bacterial interactions (Faust and Raes 2012; Zhao et al. 2022; Qin et al. 2024).

Our study confirmed that differences in heterotrophic community composition were associated with the degradation of the cyanobacterial outer layer and intracellular substances during cyanobacterial cell lysis. We emphasise the importance of incorporating algal organic matter fraction‐dependent bacterial properties and their linkages with DOM composition into quantitative models for algal‐derived DOM during the collapse of cyanobacterial blooms in Lake Taihu. Therefore, future studies should consider algal organic matter fraction‐dependent bacterial properties and their associations with key model parameters under different environmental conditions (including temperature, pH, and cyanobacterial cell densities) to enhance our understanding of the underlying mechanisms.

Our study is among the first to investigate the succession of bacterial communities in response to the addition of different algal organic matter fractions. Distinct bacterial taxa were involved in the transformation of organic carbon and the regeneration of inorganic nutrients following cyanobacterial cell lysis. The degradation of algal residual exudative organic matter (AREOM), representing DOM derived from the cyanobacterial outer layer, and algal‐derived filtrate (DOM derived from intracellular substances) led to stage‐specific changes in the chemical properties of lake water. Particle‐attached bacteria exhibited greater sensitivity than free‐living bacteria to the influence of different algal organic matter fractions over long‐term ecological observations. However, interactions among free‐living bacteria during AREOM degradation were more complex than those observed during the degradation of algal‐derived filtrate. As climate change and anthropogenic eutrophication continue to affect the distribution and frequency of cyanobacterial bloom outbreaks and decomposition in lakeshore regions, the increasing input of cyanobacterial organic matter may alter the lake carbon cycle and impact ecosystem functioning in Lake Taihu.

## Author Contributions

**Jing Chen:** conceptualization (lead); data curation (lead); formal analysis (equal); visualisation (equal); writing – original draft (lead); writing – review and editing (lead); funding acquisition (lead); project administration (lead). **Yongqiang Zhou:** investigation (equal); writing – review and editing (equal). **Yunlin Zhang:** methodology (lead); resources (lead); writing – review and editing (equal). **Quanzhong Guo:** writing – review and editing (supporting). **Shulan Zhang:** writing – review and editing (supporting). **Guanghuan Ge:** writing – review and editing (supporting). **Wenting Jin:** writing – review and editing (supporting).

## Conflicts of Interest

The authors declare no conflicts of interest.

## Supporting information


**Data S1.** Supporting Information.
**FIGURE S1.** Variations of the chlorophyll a concentrations and cyanobacterial cell densities from 2023 and 2024 (A) and sampling location (B) in Lake Taihu, China (Station 1: Wulihuxin Station; Station 2: Tuoshan Station; Station 3: Lanshanzui Station).
**FIGURE S2.** Periodic changes in the main components (C1(A), C2(B), C3(C) and C4(D)) of FDOM during the 61‐day microbial degradation of algal‐derived filtrate and algal residual exudative organic matter groups. The entire degradation could be divided into two stages: Stage I (0–20 days) and Stage II (40–61 days). Different asterisk letters indicate significant differences between experimental treatments or time stages based on one‐way ANOVA (*indicates *p* < 0.05; ** indicates *p* < 0.01; *** indicates *p* < 0.001).
**FIGURE S3.** Variations in free‐living (A) and particle‐attached (B) bacterial communities at the family level during the 61‐day microbial degradation of algal‐derived filtrate and algal residual exudative organic matter groups. Others: sum of taxa with relative abundances < 1%. The entire process can be divided into two stages: I (0–20 days) and II (40–61 days).
**FIGURE S4.** Variations of partial free‐living and particle attached bacteria at the family level during the 61‐day cyanobacterial organic matter (~1.5 g L^−1^ fresh weight) degradation.
**FIGURE S5.** Venn diagrams on the ASV level in free‐living bacterial communities of the algal‐derived filtrate (AF) group and algal residual exudative organic matter (AREOM) group at stage I (a) and stage II (b); venn diagrams on the ASV level in particle‐attached bacterial communities of the AF and AREOM group at stage I (c) and stage II (d).
**FIGURE S6.** Co‐occurrence networks of the dominant free‐living (FL) or particle‐attached (PA) bacterial families at stage I and stage II of the algal‐derived filtrate [FL‐I (A); FL‐II (B); PA‐I (C); PA‐II (D)] and algal residual exudative organic matter [FL‐I (E); FL‐II (F); PA‐I (G); PA‐II (H)] groups, with a Spearman’s coefficient threshold of 0.7 and an adjusted *p*‐value threshold of 0.05 throughout the degradation period. Node colours indicate different modularity classes. The size of each node is proportional to the number of degrees.

## Data Availability

Data sharing not applicable to this article as no datasets were generated or analysed during the current study.
