# Supplementary material for: Succession of Particle‐Attached and Free‐Living Microbial Communities in Response to the Degradation of Algal Organic Matter in Lake Taihu, China
Source: Environ Microbiol Rep. 2025 Apr 20;17(2):e70094. doi: 10.1111/1758-2229.70094 (PMC12009638; doi:10.1111/1758-2229.70094)
Supplement: Supplementary file 1 — Data S1. Supporting Information. FIGURE S1. Variations of the chlorophyll a concentrations and cyanobacterial cell densities from 2023 and 2024 (A) and sampling location (B) in Lake Taihu, China (Station 1: Wulihuxin Station; Station 2: Tuoshan Station; Station 3: Lanshanzui Station). FIGURE S2. Periodic changes in the main components (C1(A), C2(B), C3(C) and C4(D)) of FDOM during the 61‐day microbial degradation of algal‐derived filtrate and algal residual exudative organic matter groups. The entire degradation could be divided into two stages: Stage I (0–20 days) and Stage II (40–61 days). Different asterisk letters indicate significant differences between experimental treatments or time stages based on one‐way ANOVA (*indicates p < 0.05; ** indicates p < 0.01; *** indicates p < 0.001). FIGURE S3. Variations in free‐living (A) and particle‐attached (B) bacterial communities at the family level during the 61‐day microbial degradation of algal‐derived filtrate and algal residual exudative organic matter groups. Others: sum of taxa with relative abundances < 1%. The entire process can be divided into two stages: I (0–20 days) and II (40–61 days). FIGURE S4. Variations of partial free‐living and particle attached bacteria at the family level during the 61‐day cyanobacterial organic matter (~1.5 g L−1 fresh weight) degradation. FIGURE S5. Venn diagrams on the ASV level in free‐living bacterial communities of the algal‐derived filtrate (AF) group and algal residual exudative organic matter (AREOM) group at stage I (a) and stage II (b); venn diagrams on the ASV level in particle‐attached bacterial communities of the AF and AREOM group at stage I (c) and stage II (d). FIGURE S6. Co‐occurrence networks of the dominant free‐living (FL) or particle‐attached (PA) bacterial families at stage I and stage II of the algal‐derived filtrate [FL‐I (A); FL‐II (B); PA‐I (C); PA‐II (D)] and algal residual exudative organic matter [FL‐I (E); FL‐II (F); PA‐I (G); PA‐II (H)] groups, with [file EMI4-17-e70094-s001.doc]

**Title:**

Succession of particle-attached and free-living microbial communities in response to the degradation of algal organic matter in Lake Taihu, China

Jing Chen ^1,2,3^ **|** Yongqiang Zhou ^2,3,*^ **|** Yunlin Zhang ^2,3^ **|** Quanzhong Guo ^1^ **|** Shulan Zhang ^1^ **|** Guanghuan Ge ^1^**|** Wenting Jin ^1^

^1^ College of Chemistry and Environment, Ankang University, Ankang, Shaanxi, 725000, China

^2^ Nanjing Institute of Geography and Limnology, Chinese Academy of Sciences, Nanjing, 211135, China

^3^ University of Chinese Academy of Sciences, Beijing, 100049, China

First author: [chenjing@aku.edu.cn](mailto:chenjing@aku.edu.cn) (J. Chen)

*Correspondence: [yqzhou@niglas.ac.cn](mailto:yqzhou@niglas.ac.cn) (Y. Zhou)

Text

Text S1: Analysis of DOC concentration and main components of Fluorescent Dissolved Organic Matter (FDOM)

Concentrations of DOC: The DOC concentrations were measured by a TOC-L analyzer with an ASI-L autosampler (Shimadzu, TOC-L CPH, Japan) through high-temperature catalytic oxidation (HTCO) (Benner & Strom, 1993). Potassium hydrogen phthalate solution was used as the analytical DOC standard, and it was determined to maintain data quality control (Chen et al., 2022). The blank was deducted using Milli-Q water analysis before every five samples, and all samples (including the blank) were acidified to pH = 2 by 10% HCl until the analysis. The average blanks associated with the DOC measurement were approximately 0.06 mg/L, and the analytic precision of the triplicate injections was ±3%.

Main components of FDOM: The main components of FDOM were analyzed by fluorescence spectroscopy technique coupled with parallel factor (PARAFAC) analysis (Zhang et al., 2009; Li et al., 2018). A fluorescence spectrophotometer (Hitachi F-7000) with a scanning speed of 2400 nm/min was employed to measure the excitation-emission matrix (EEM). The EEM fluorescence spectra was gathered with scanning emission (Em) wavelength from 250 to 600 nm at 1 nm increments, and the excitation (Ex) wavelength from 200 to 450 nm at 5 nm increments, respectively. The blank scans were performed every 10 analyses using Milli-Q water. And Rayleigh and Raman scattering (peak emission ±10–15 nm at each excitation wavelength) were removed from the EEM spectra, and then filled in the missing regions by the three-dimensional Delaunay interpolation of the surrounding data points (Barber et al., 1996). The chromophoric DOM absorbance was used to correct the measured EEMs to eliminate the inner-filter effect, as described in previous studies (Zhang et al., 2010; Zhang et al., 2013). CDOM absorbance was measured over the 200–800 nm range (1 nm increments) in a 5-cm quartz cell using a Shimadzu UV-2450 PC UV–vis recording spectrophotometer. During the experiments, a total of 42 EEMs spectra were obtained for PARAFAC analysis. MATLAB (MathWorks, Natick, MA) together with the DOM Fluor toolbox (http://www.models.life. ku.dk/) were employed for data analysis (Coble 1996; Coble et al., 1998).

Text S2: Analysis of bacterial community structure

Prior to bacterial community structure analysis, three parallel samples at each sampling time point were subjected to bacterial DNA extraction. Concrete extraction was carried out according to the phenol-chloroform-isoamyl alcohol method (Harder et al., 2003). The final DNA quality and purity were determined by 1% agarose gel electrophoresis. The 16S rRNA gene was amplified with adapter primers targeting the V3-V4 hypervariable regions: 341F (5’-CCTAYGGGRBGCASCAG-3’) and 806R (5’-GGACTACNNGGGTATCTAAT-3’) using a PCR system (T100PCR, Bio-Rad, USA). All PCR reactions were carried out with 15 µL of Phusion® High-Fidelity PCR Master Mix (New England Biolabs), 2 µM of forward and reverse primers, and approximately 10 ng template DNA. The PCR procedures were as follows: initial denaturation at 98℃ for 1 min, followed by 30 cycles of denaturation at 98℃ for 10 s, annealing at 50℃ for 30 s, and elongation at 72℃ for 30 s, extension at 72 ℃ for 5 min. The same volume of 1XTAE buffer was mixed with the PCR products, and electrophoresis was conducted on 2% agarose gel for detection. The PCR products were mixed at equidensity ratios. PCR products were purified using a Qiagen Gel Extraction Kit (Qiagen, Germany) (Shvartsman et al., 2022). Paired-end (2 × 250 bp) sequencing was performed on an Illumina NovaSeq platform (Illumina, San Diego, CA, USA) by Wekemo Tech Group Co., Ltd. (Shenzhen, China) (Zhai et al., 2024), then 250 bp paired-end reads were generated. The barcode and connector sequence were cleared away, and the double-ended sequences were stitched by using FLASH (v1.2.8) and the unqualified sequences were filtered out (Zhu et al., 2022). Raw sequence data were deposited in the Genome Sequence Archive (Genomics, Proteomics & Bioinformatics 2021) at the National Genomics Data Center (Nucleic Acids Res 2022), China National Center for Bioinformation (Accession Number: CRA017057). Data analyses were performed using QIME2 software. Finally, sequences were aligned to the Greengenes 13_8 99% Amplicon Sequence Variant (ASV) database after gene quality filtering (Mohamed et al., 2023). A taxonomy table was generated to analyze the relative abundance of free-living and attached bacteria at the class and family levels among both groups.

References

Barber, C.B., Dobkin, D.P. & Huhdanpaa, H.T. (1996) The quick hull algorithm for convex hulls. *ACM Transactions on Mathematical Software*, 22(4), 469-483. Available from: <https://doi.org/10.1145/235815.235821>

Benner, R*.* & Strom, M. (1993) A critical evaluation of the analytical blank associated with DOC measurements by high-temperature catalytic oxidation. *Marine Chemistry*, 41(1-3), 153-160. Available from: <https://doi.org/10.1016/0304-4203(93)90113-3>

Chen, J., Zhou, Y.Q. & Zhang, Y.L. (2022) New Insights into Microbial Degradation of Cyanobacterial Organic Matter Using a Fractionation Procedure. *International Journal of Environmental Research and Public Health*, 19, 6981. Available from: <https://doi.org/10.3390/ijerph19126981>

Coble, P.G. (1996) Characterization of marine and terrestrial DOM in seawater using excitation-emission matrix spectroscopy. *Marine Chemistry*, 51(4), 325-346. Available from: <https://doi.org/10.1016/0304-4203(95)00062-3>

Coble, P.G., Castillo, C.E.D. & Avril, B. (1998) Distribution and optical properties of CDOM in the Arabian Sea during the 1995 Southwest Monsoon. *Deep Sea Research Part II*, 45(10-11), 2195-2223. Available from: <https://doi.org/10.1016/S0967-0645(98)00068-X>

Harder, T., Lau, S.C.K., Dobretsov, S., Fang, T.K. & Qian, P.-Y. (2003) A distinctive epibiotic bacterial community on the soft coral *Dendronephthya* sp. and antibacterial activity of coral tissue extracts suggest a chemical mechanism against bacterial epibiosis. *FEMS Microbiology Ecology*, 43(3), 337-347. Available from: <https://doi.org/10.1016/S0168-6496(02)00434-8>

Li, H.M., Zhang, Y.Y., Liang, Y.T., Chen, J., Zhu, Y.C., Zhao, Y.T. et al. (2018) Impacts of mar- icultural activities on characteristics of dissolved organic carbon and nutrients in a typical raft-culture area of the Yellow Sea, North China. *Marine Pollution Bulletin*, 137, 456-464. Available from: <https://doi.org/10.1016/j.marpolbul.2018.10.048>

Mohamed, H.F., Abd‑Elgawad, A., Cai, R.S., Luo, Z.H., Pie, L.L. & Xu, C.A. (2023) Microbial community shift on artificial biological reef structures (ABRs) deployed in the South China Sea. Scientific Reports, 13, 3456. Available from: <https://doi.org/10.1038/s41598-023-29359-5>

Shvartsman, E., Richmond, M.E.I., Schellenberg, J.J., Lamont, A., Perciani, C., Russell, J.N.H. et al. (2022) Comparative analysis of DNA extraction and PCR product purification methods for cervicovaginal microbiome analysis using *cpn60* microbial profiling. *PLoS ONE*, 17(1), e0262355. Available from: <https://doi.org/10.1371/journal.pone.0262355>

Zhai, C.C., Han, L.L., Xiong, C., Ge, A.H., Yue, X.J., Li, Y. et al. (2024) Soil microbial diversity and network complexity drive the ecosystem multifunctionality of temperate grasslands under changing precipitation. *Science of The Total Environment*, 906, 167217. Available from: <https://doi.org/10.1016/j.scitotenv.2023.167217>

Zhang, Y.L., Dijk, M.A., Liu, M.L., Zhu, G.W. & Qin, B.Q. (2009) The contribution of phytoplankton degradation to chromophoric dissolved organic matter (CDOM) in eutrophic shallow lakes: field and experimental evidence. *Water Research*, 43(18), 4685-4697. Available from: <https://doi.org/10.1016/j.watres.2009.07.024>

Zhang, Y.L., Liu, X.H., Wang, M.Z. & Qin, B.Q. (2013) Compositional differences of chromophoric dissolved organic matter derived from phytoplankton and macrophytes. *Organic Geochemistry*, 55(1), 26-37. Available from: <https://doi.org/10.1016/j.orggeochem.2012.11.007>

Zhang, Y.L., Zhang, E.L., Yin, Y., Dijk, M.A., Feng, L.Q., Shi, Z.Q. et al. (2010) Characteristics and sources of chromophoric dissolved organic matter in lakes of the Yungui Plateau, China, differing in trophic state and altitude. *Limnology and Oceanography*, 55(6), 2645-2659. Available from: <https://doi.org/10.4319/lo.2010.55.6.2645>

Zhu, H.K., Wang, R.Y., Hua, H.Y., Cheng, Y.L., Guo, Y.H., Qian, H. et al. (2022) Network Pharmacology Exploration Reveals Gut Microbiota Modulation as a Common Therapeutic Mechanism for Anti-Fatigue Effect Treated with Maca Compounds Prescription. *Nutrients*, 14(8), 1533. Available from: <https://doi.org/10.3390/nu14081533>

Figure


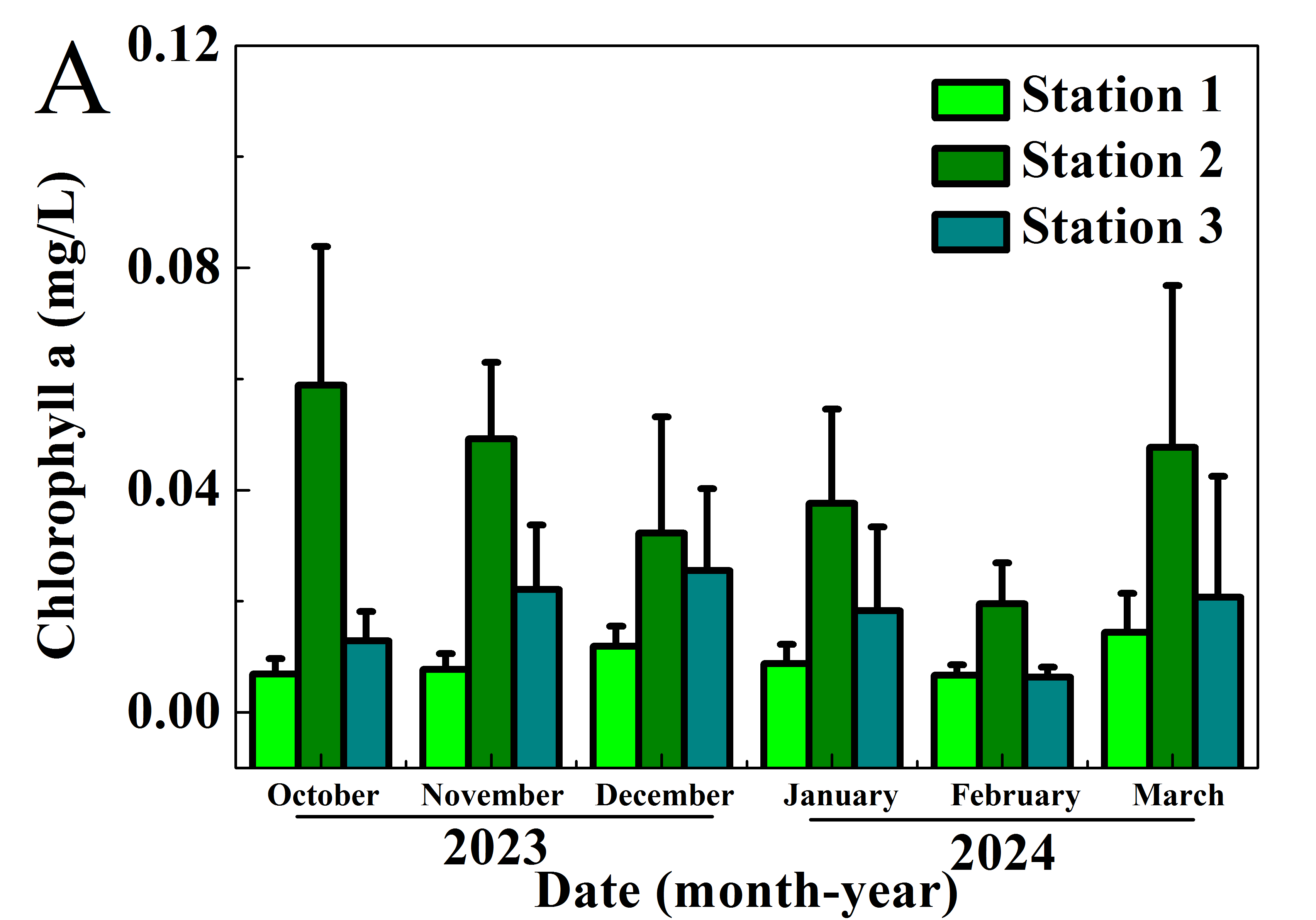

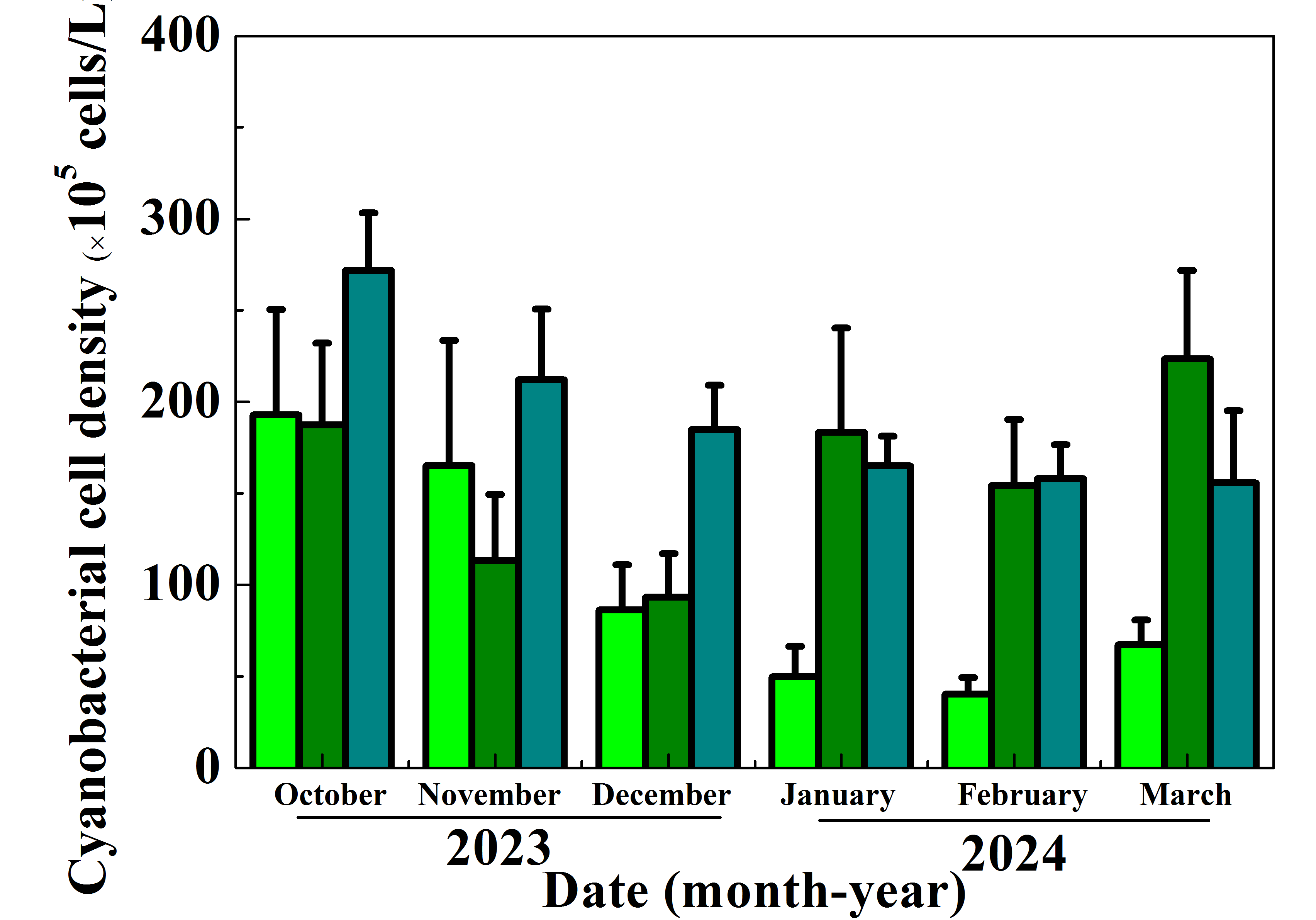


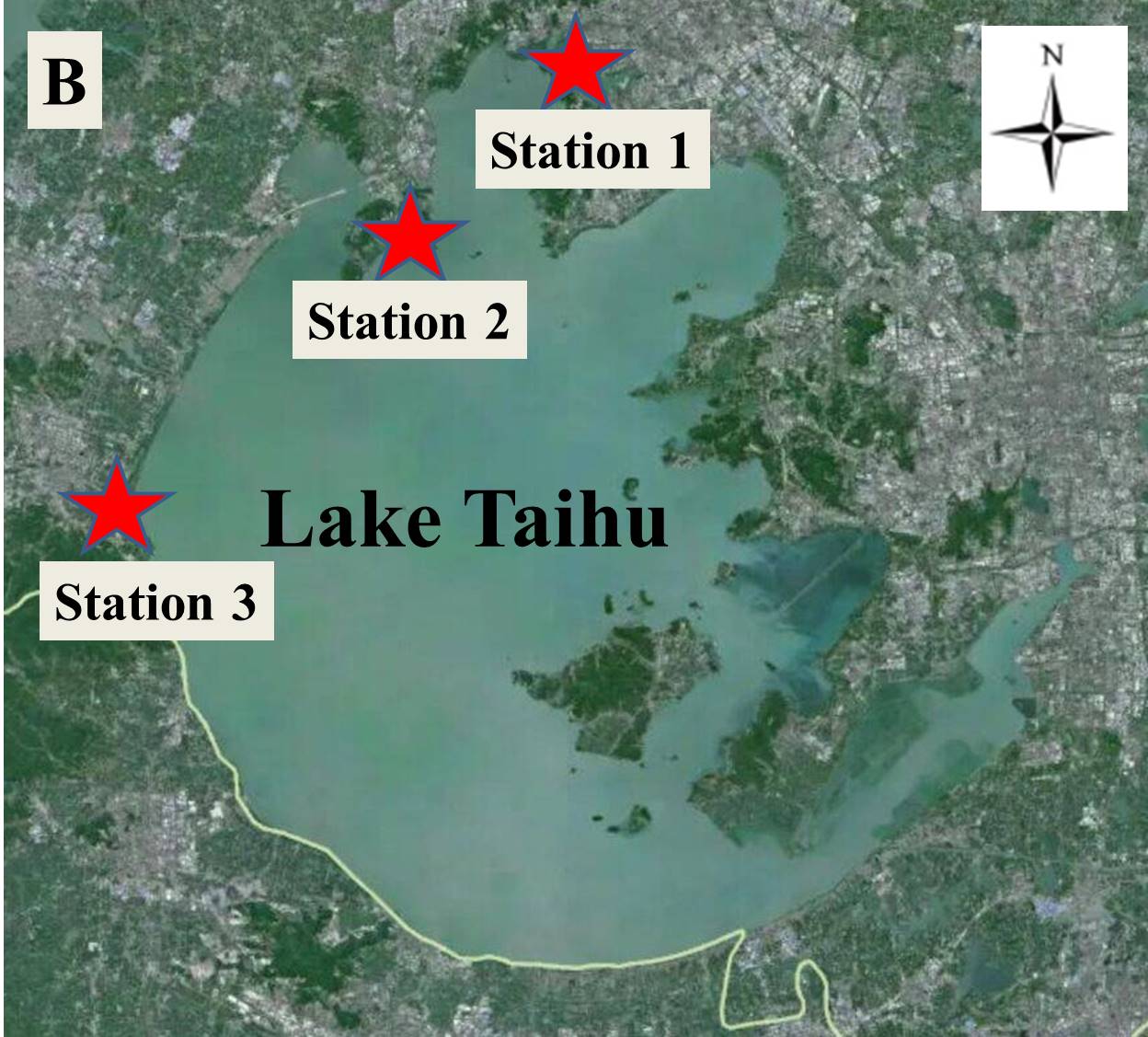


**FIGURE S1** Variations of the chlorophyll a concentrations and cyanobacterial cell densities from 2023 and 2024 (A) and sampling location (B) in Lake Taihu, China (Station 1: Wulihuxin Station; Station 2: Tuoshan Station; Station 3: Lanshanzui Station).


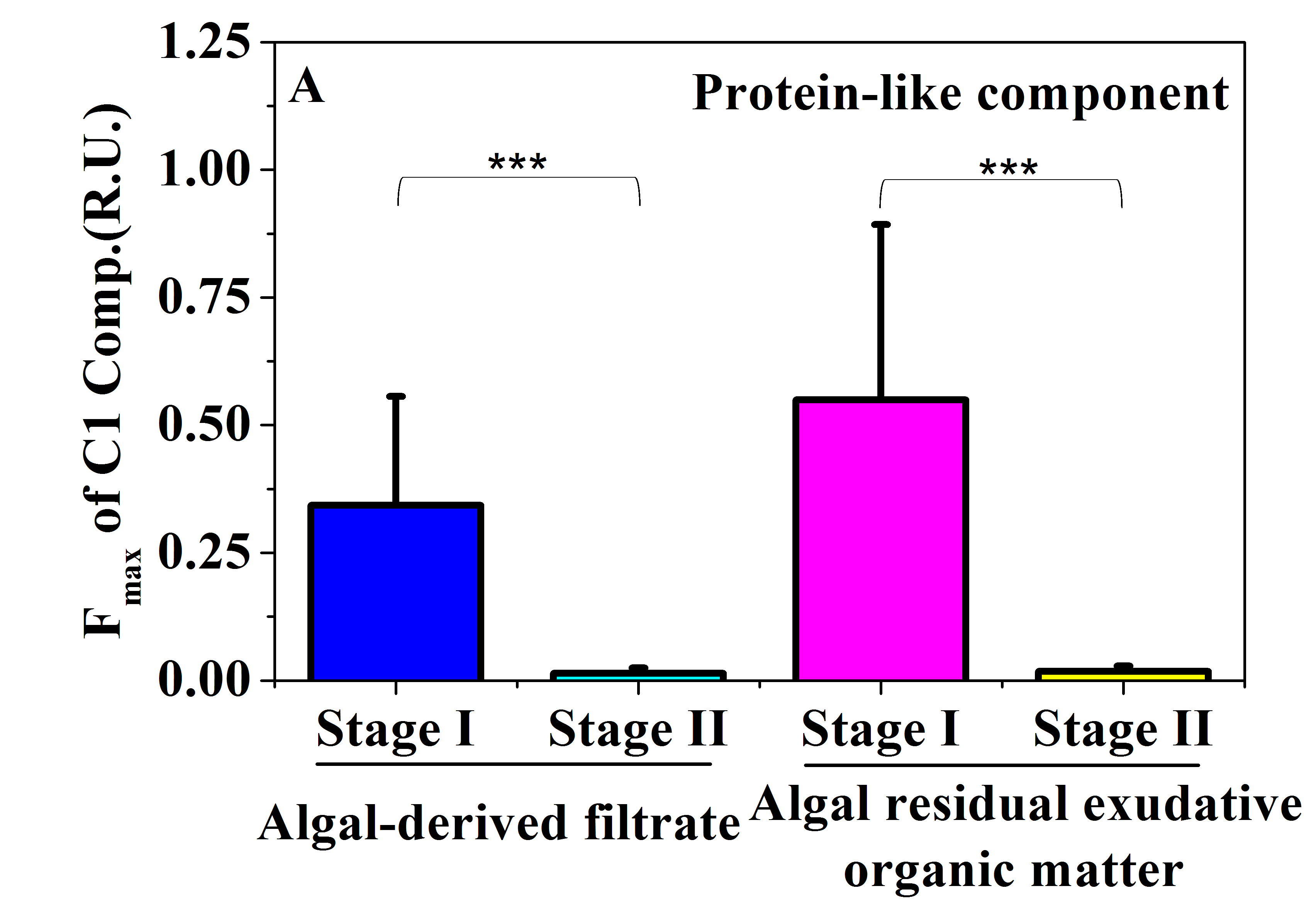

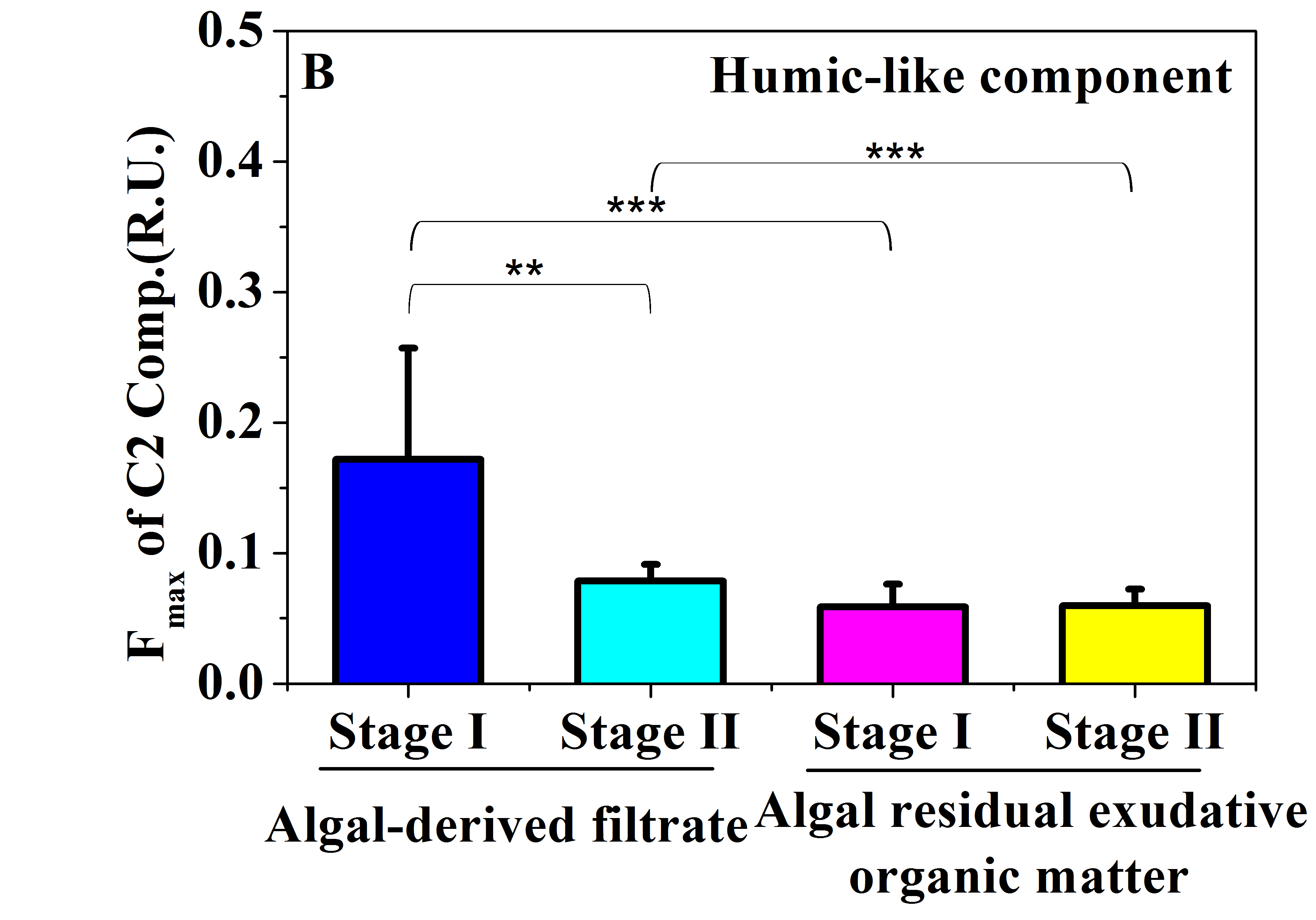


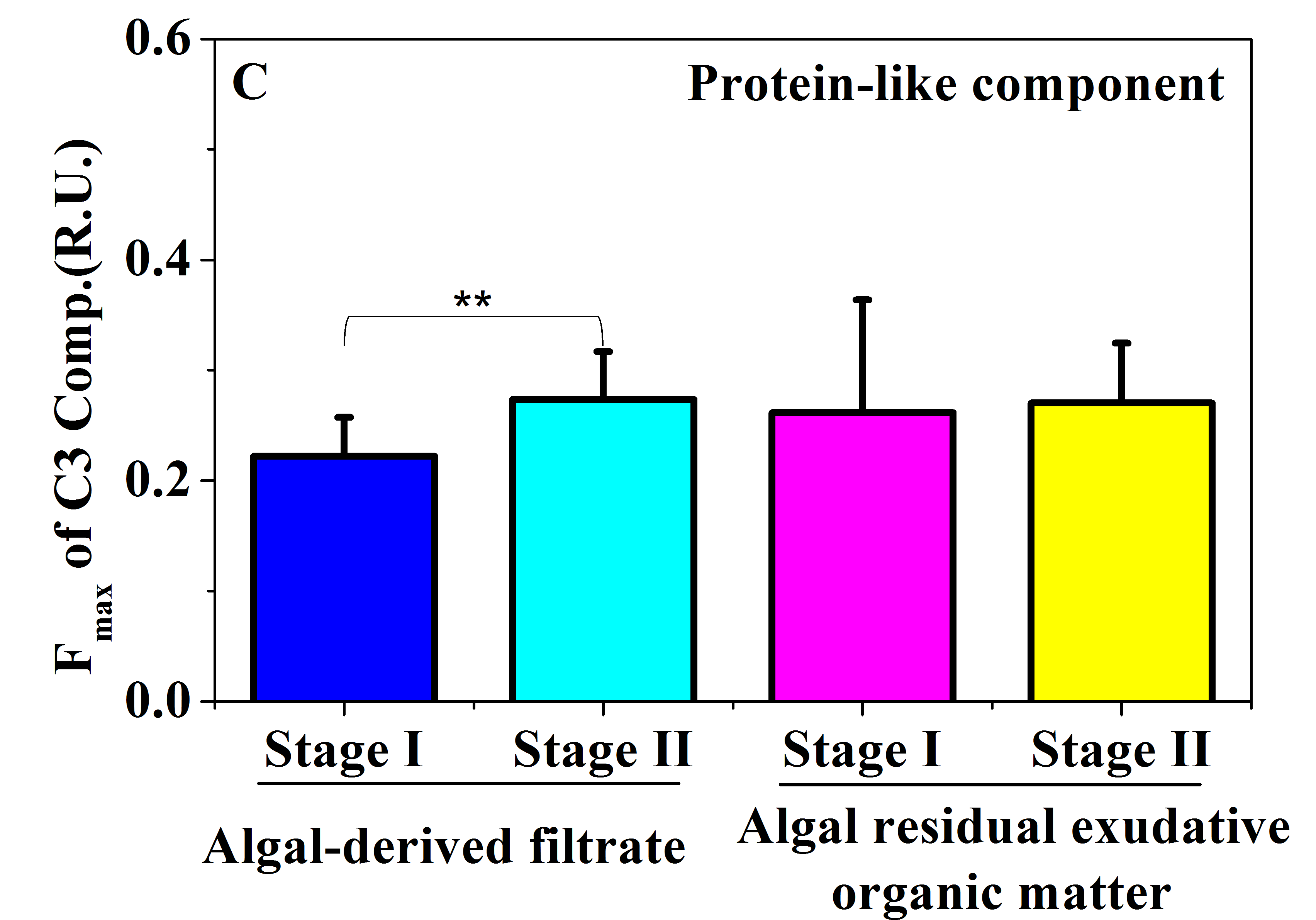

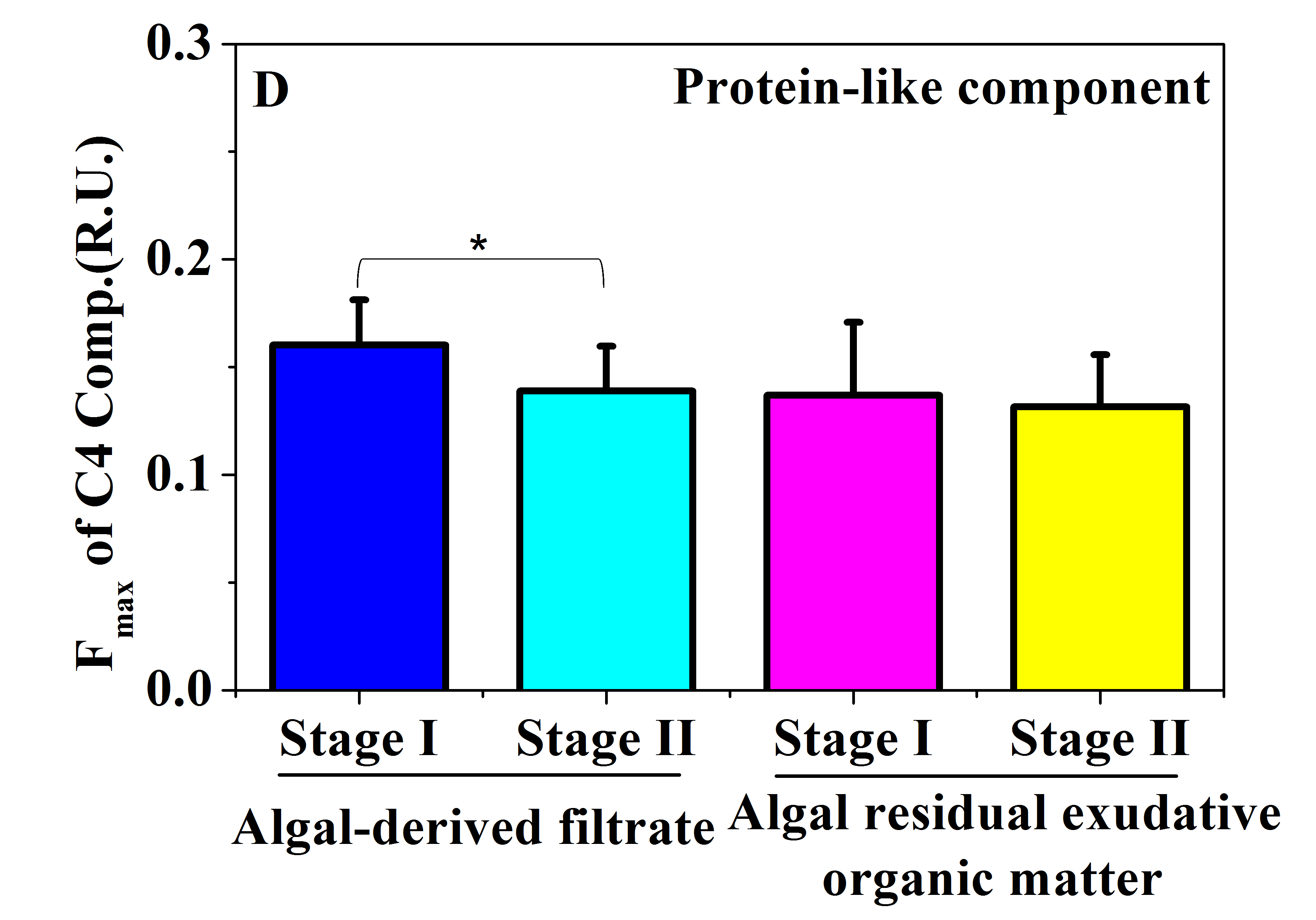


**FIGURE S2** Periodic changes in the main components (C1(A), C2(B), C3(C) and C4(D)) of FDOM during the 61-day microbial degradation of algal-derived filtrate and algal residual exudative organic matter groups. The entire degradation could be divided into two stages: Stage I (0–20 days) and Stage II (40–61 days). Different asterisk letters indicate significant differences between experimental treatments or time stages based on one-way ANOVA (*indicates *p* < 0.05; ** indicates *p* < 0.01; *** indicates *p* < 0.001).

**FIGURE S3** Variations in free-living (A) and particle-attached (B) bacterial communities at the family level during the 61-day microbial degradation of algal-derived filtrate and algal residual exudative organic matter groups. Others: sum of taxa with relative abundances < 1%. The entire process can be divided into two stages: I (0–20 days) and II (40–61 days).


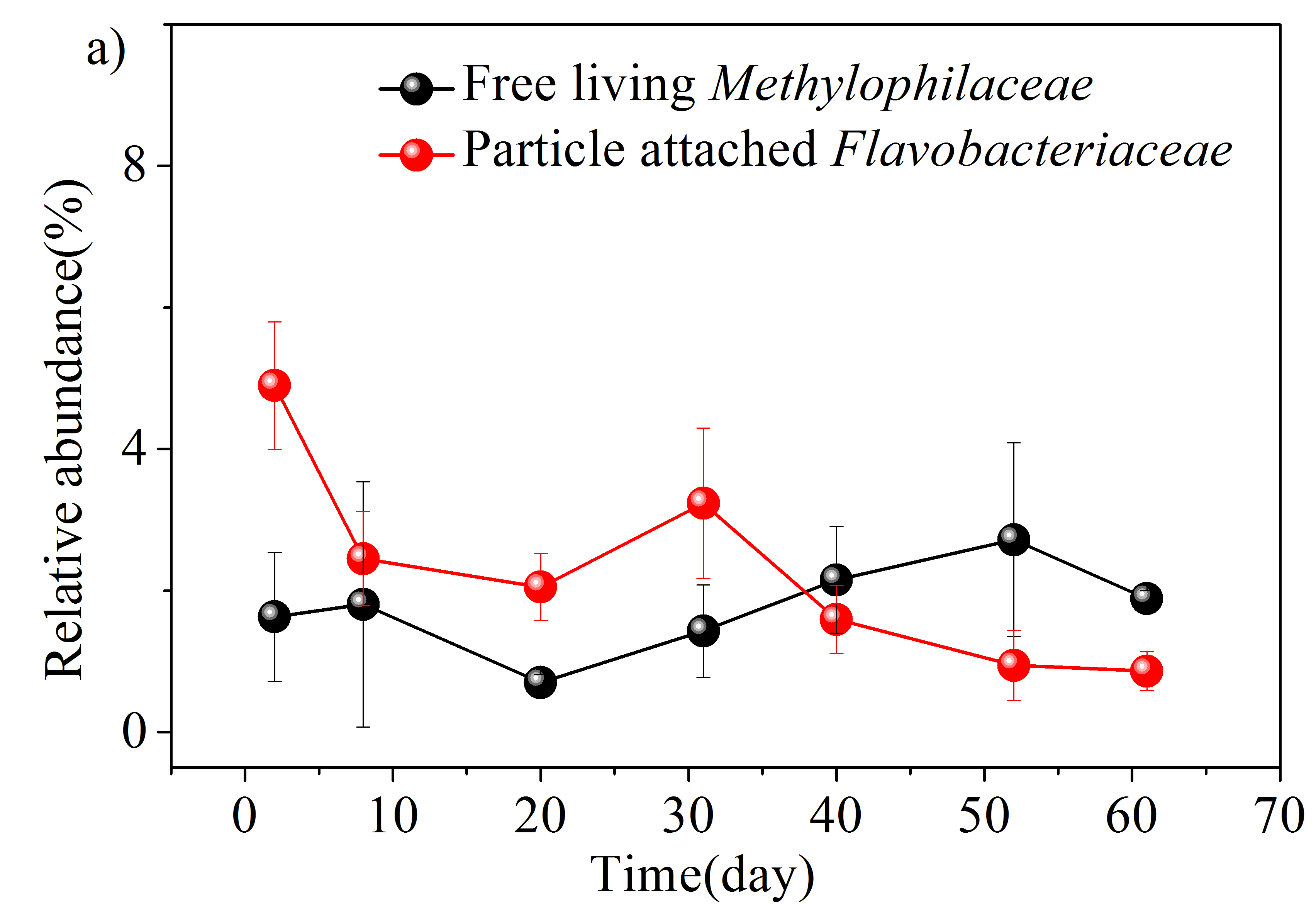

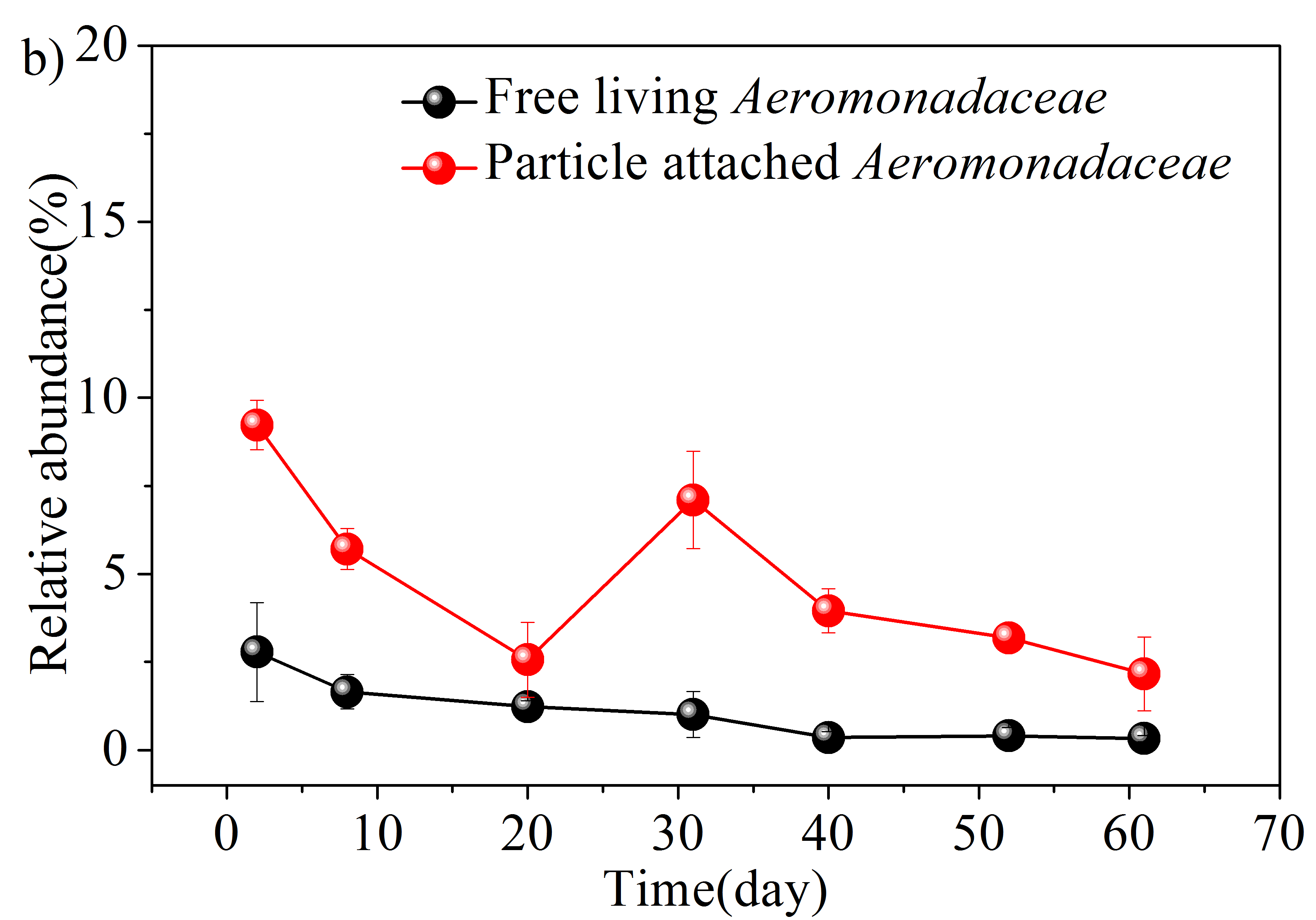


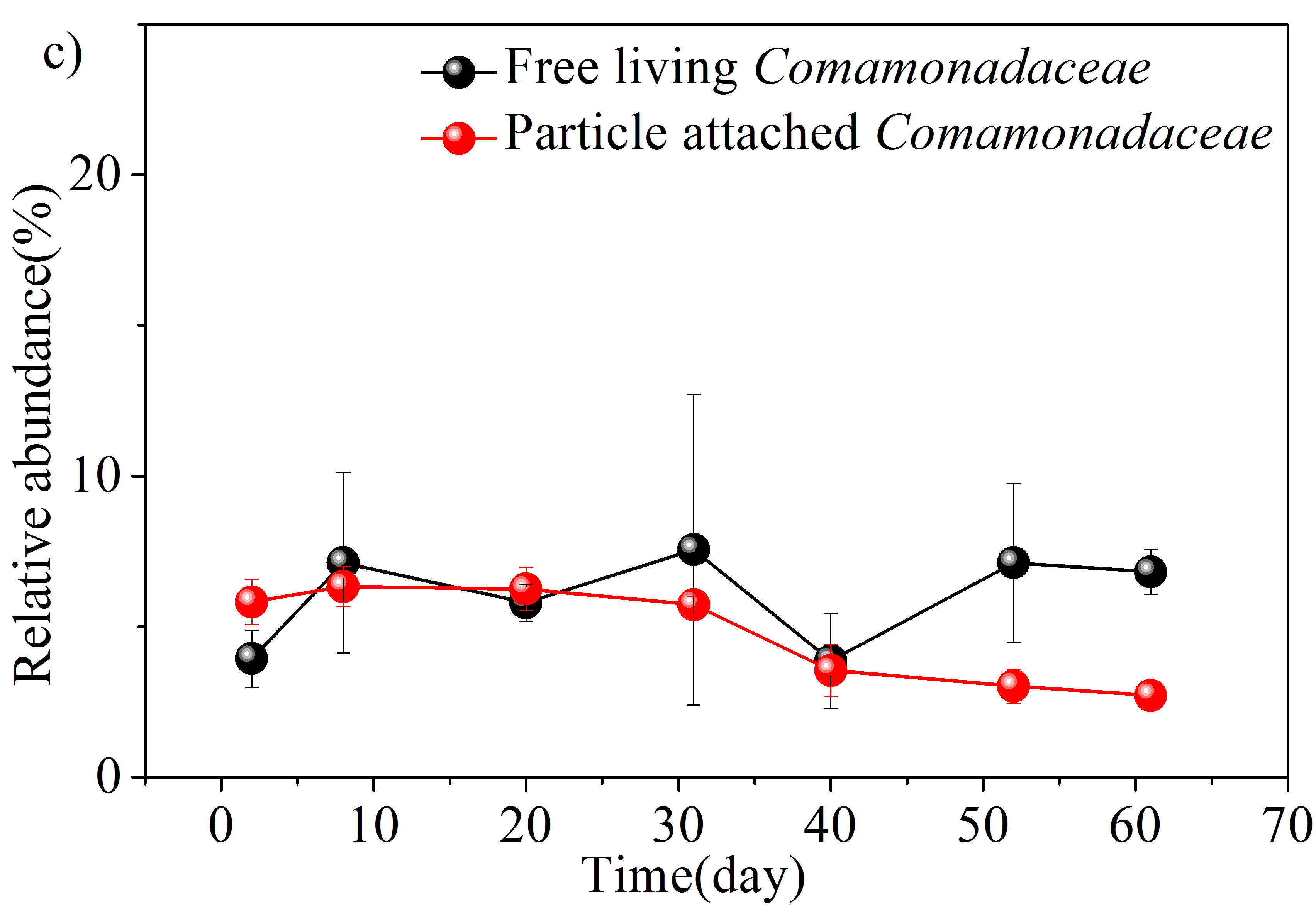

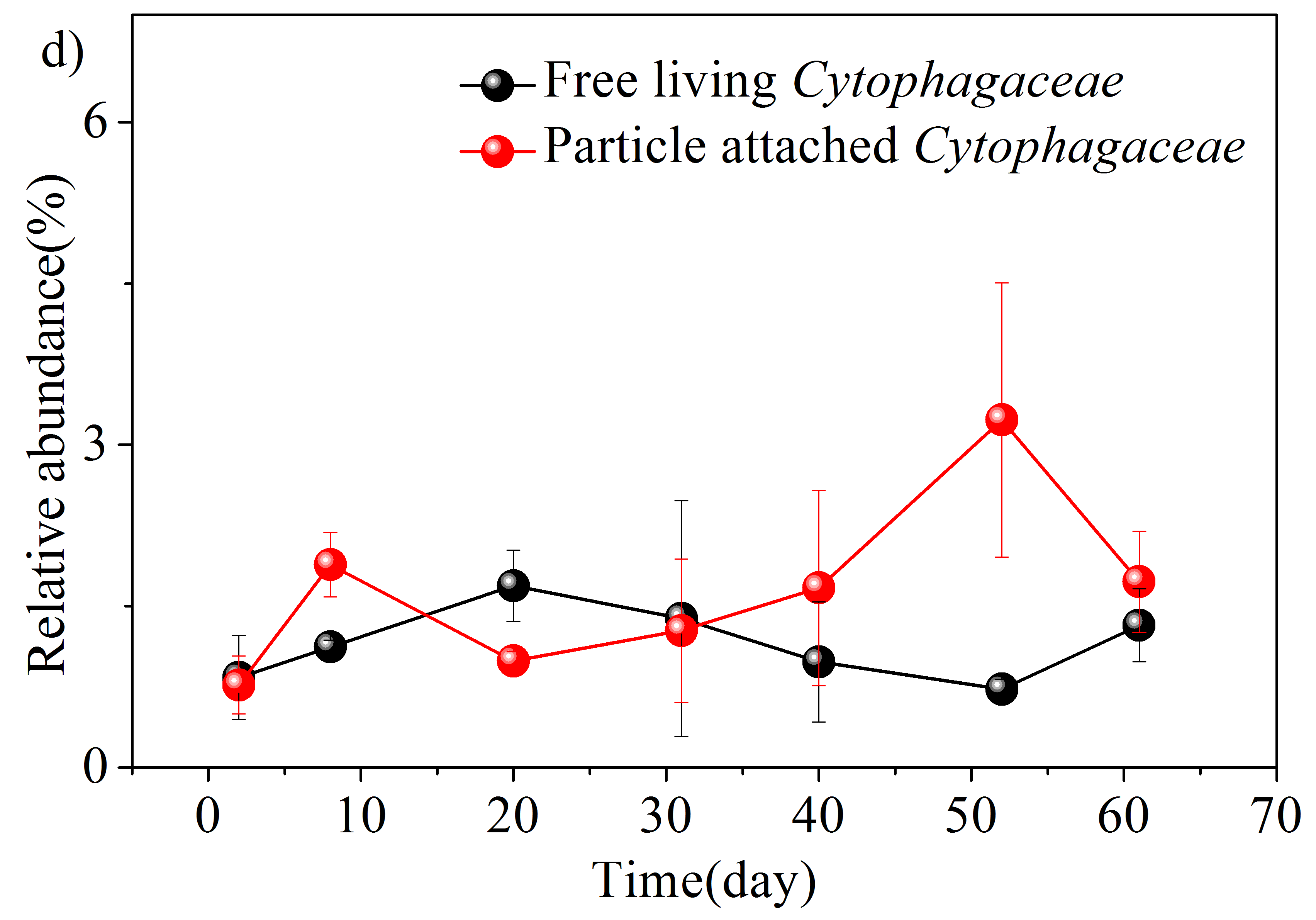


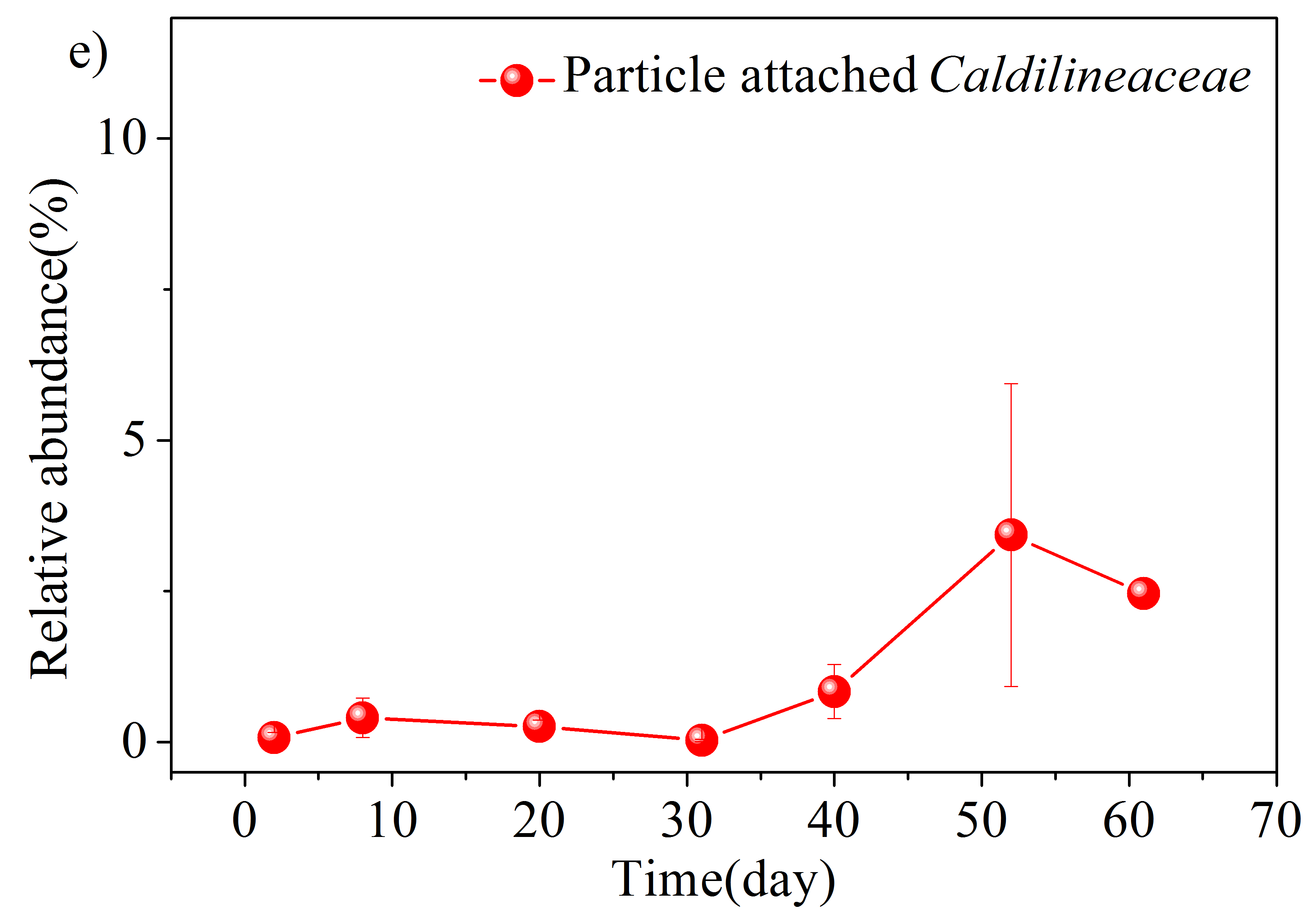


**FIGURE S4** Variations of partial free-living and particle attached bacteria at the family level during the 61-day cyanobacterial organic matter (~1.5 g L^-1^ fresh weight) degradation.

**FIGURE S5** Venn diagrams on the ASV level in free-living bacterial communities of the algal-derived filtrate (AF) group and algal residual exudative organic matter (AREOM) group at stage I (a) and stage II (b); venn diagrams on the ASV level in particle-attached bacterial communities of the AF and AREOM group at stage I (c) and stage II (d).

**FIGURE S6** Co-occurrence networks of the dominant free-living (FL) or particle-attached (PA) bacterial families at stage I and stage II of the algal-derived filtrate [FL-I (A); FL-II (B); PA-I (C); PA-II (D)] and algal residual exudative organic matter [(FL-I (E); FL-II (F); PA-I (G); PA-II (H)] groups, with a Spearman’s coefficient threshold of 0.7 and an adjusted *p*-value threshold of 0.05 throughout the degradation period. Node colors indicate different modularity classes. The size of each node is proportional to the number of degrees.
